# Deconvolution of the Response to Bacillus Calmette–Guérin Reveals NF-κB-Induced Cytokines As Autocrine Mediators of Innate Immunity

**DOI:** 10.3389/fimmu.2017.00796

**Published:** 2017-07-13

**Authors:** Aurélie Bisiaux, Jeremy Boussier, Darragh Duffy, Lluis Quintana-Murci, Magnus Fontes, Matthew L. Albert, The Milieu Intérieur Consortium

**Affiliations:** ^1^INSERM U1223, Paris, France; ^2^Laboratory of Dendritic Cell Immunobiology, Department of Immunology, Institut Pasteur, Paris, France; ^3^International Group for Data Analysis, Institut Pasteur, Paris, France; ^4^Laboratory of Human Evolutionary Genetics, Department of Genomes and Genetics, Institut Pasteur, Paris, France; ^5^CNRS URA3012, Paris, France; ^6^Department of Immunology, Genentech Inc., South San Francisco, CA, United States; ^7^Institut Pasteur, Paris, France

**Keywords:** mycobacteria, immune monitoring, toll-like receptors, Dectin-1/2, Jak-1/2 inhibitors

## Abstract

Bacillus Calmette–Guérin (BCG) is used as a vaccine and diagnostic test for tuberculosis, as well as immunotherapy in the treatment of bladder cancer. While clinically useful, the response to mycobacterial stimulation is complex and the induced protein signature remains poorly defined. We characterized the cell types directly engaged by BCG, as well as the induced cytokine loops that transmit signal(s) to bystander cells. Standardized whole-blood stimulations and mechanistic studies on single and purified cell populations identified distinct patterns of activation in monocytes as compared to neutrophils and invariant lymphocyte populations. Deconvoluting the role of Toll-like receptor 2/4 and Dectin-1/2 in the inflammatory response to BCG, we revealed Dectin-1/2 as dominant in neutrophils as compared to monocytes, which equally engaged both pathways. Furthermore, we quantified the role of NF-κB and NADPH/reactive oxygen species (ROS)-dependent cytokines, which triggered a JAK1/2-dependent amplification loop and accounted for 40–50% of the induced response to BCG. In sum, this study provides new insight into the molecular and cellular pathways involved in the response to BCG, establishing the basis for a new generation of immunodiagnostic tools.

## Introduction

The bacillus Calmette–Guérin (BCG) vaccine strain has been used for the prevention of childhood tuberculosis (TB) for more than 90 years. It is one of the most widely used vaccines, delivered to >80% of neonates and infants worldwide (1). Following from the seminal work of Morales et al. in 1976 (2), BCG is also the treatment of choice for non-muscle invasive bladder cancer (3). However, there remains a need for a clinical test to predict an individual’s response to intravesical BCG treatment given that 30–50% of patients do not respond (3, 4). In addition, there is a lack of an effective blood based diagnostic tool to support the management of infected individuals with TB. One advance has been the introduction of two *ex vivo* interferon gamma release assays (IGRAs) to replace the use of the purified peptide derivative skin test (5). Both IGRAs are assessed based on IFN-γ production: the number of TB antigen-specific T cells producing IFN-γ for T-SPOT.TB (modified ELISPOT) and IFN-γ concentration for QFT (conventional ELISA). In line with these efforts, our laboratory recently described an *ex vivo* assay based on whole-blood stimulation, referred to as TruCulture assays (6). To fully harness these new tools, it is important to achieve a deeper understanding of the multiple host sensors responsible for the variable host response to mycobacterial stimulation.

Our previous study defined the boundaries of a healthy immune response to BCG, as well as other stimuli, including lipoarabinomannan (LAM) (a cell wall component of mycobacteria) and other agonists that mimic the host response to bacterial stimulation (6). These initial results suggested that the complex protein signature induced by BCG is the result of at least two factors: (i) BCG contains multiple immune agonists (7, 8) and (ii) a heterogeneous mixture of cell types, as present in whole blood, express distinct sets of host sensors and respond differentially. As an additional level of complexity, directly activated cells may convey the inflammatory response to bystander cells *via* the production of inflammatory cytokines. We hypothesized a key role for host-derived factors, notably cytokines, as critical for the broad protein signature response to BCG stimulation.

As a starting point for the current study, we proposed that the response to BCG could be understood as an integration of host sensor activation and induced cytokines that serve to transmit the inflammatory signals. We investigated the engagement of selected host receptors by BCG [e.g., the principle toll-like receptors (TLRs), TLR2 and TLR4], as well as the C-type lectin-like receptors (CLRs) Dectin-1/2, which have been reported to be involved in mycobacteria–host cells interaction (7, 9, 10), and the key innate cytokines [e.g., IL-6, tumor necrosis factor-alpha (TNF-α), and IL-1β], which may mediate a feed-forward host response. Globally, the engaged host sensors acted *via* NF-κB-mediated transcription, which was dependent upon NADPH oxidase-derived ROS. Strikingly, we identified a unique pattern of cell-type specific activation, with Dectin-1/2 engagement by BCG being dominant in neutrophils as compared to monocyte and lymphocyte populations. Finally, we observed that the initial wave of induced cytokines amplified the innate response through a combined activation of multiple cytokine receptors and subsequent JAK1/2-dependent signaling. These data will support a deeper understanding of the variable host response to mycobacteria.

## Materials and Methods

### Human Samples

**Figure 1:**
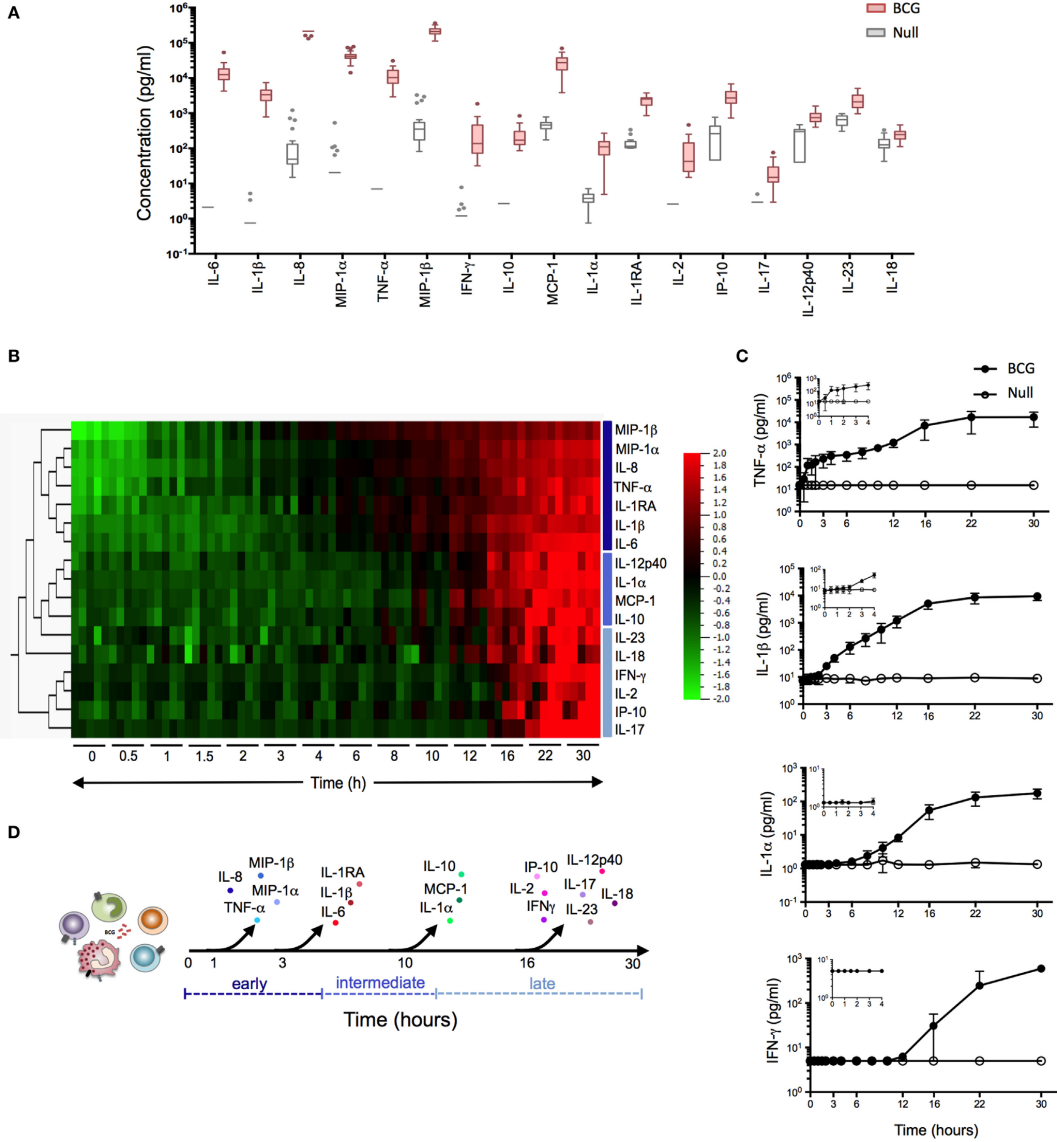
Bacillus Calmette–Guérin (BCG) stimulation induces a broad array of inflammatory proteins. **(A)** Whole blood from 25 healthy donors was incubated for 22 h in the presence of 3 × 10^5^ CFU of BCG or buffer control (null condition). The culture supernatant was assayed for the expression levels of 32 proteins (Table S1 in Supplementary Material). Tukey box-whisker plots indicate those proteins (*n* = 17) that were significantly induced by BCG stimulation as compared to the null tube, as defined by ANOVA (filtered to include analytes with *q* value < 0.01). Analytes are ordered from the highest to the lowest fold change, BCG stimulation vs. null. **(B–D)** Whole blood from five healthy donors was incubated in the presence of 3 × 10^5^ CFU of BCG or buffer control over a 30 h time course. At each time point, culture supernatants were collected and analyzed. **(B)** Heat map representation of the 17 BCG-induced proteins over the 30 h time course (0 h = pre-stimulation sample). Protein concentrations were log-transformed and ordered based on hierarchical clustering, showing the earliest (MIP-1β) to the latest (IL-17) induced protein analyte. Cytokines were color-coded based on their clustering. **(C)** Median concentration of four representative cytokines (one shown for immediate, early, intermediate, and late release) are shown, with graphical inlay plotting the first 4 h poststimulation. **(D)** Schematic representation illustrates the kinetic response for the 17 cytokines that were induced following BCG exposure.

**Figure 2:**
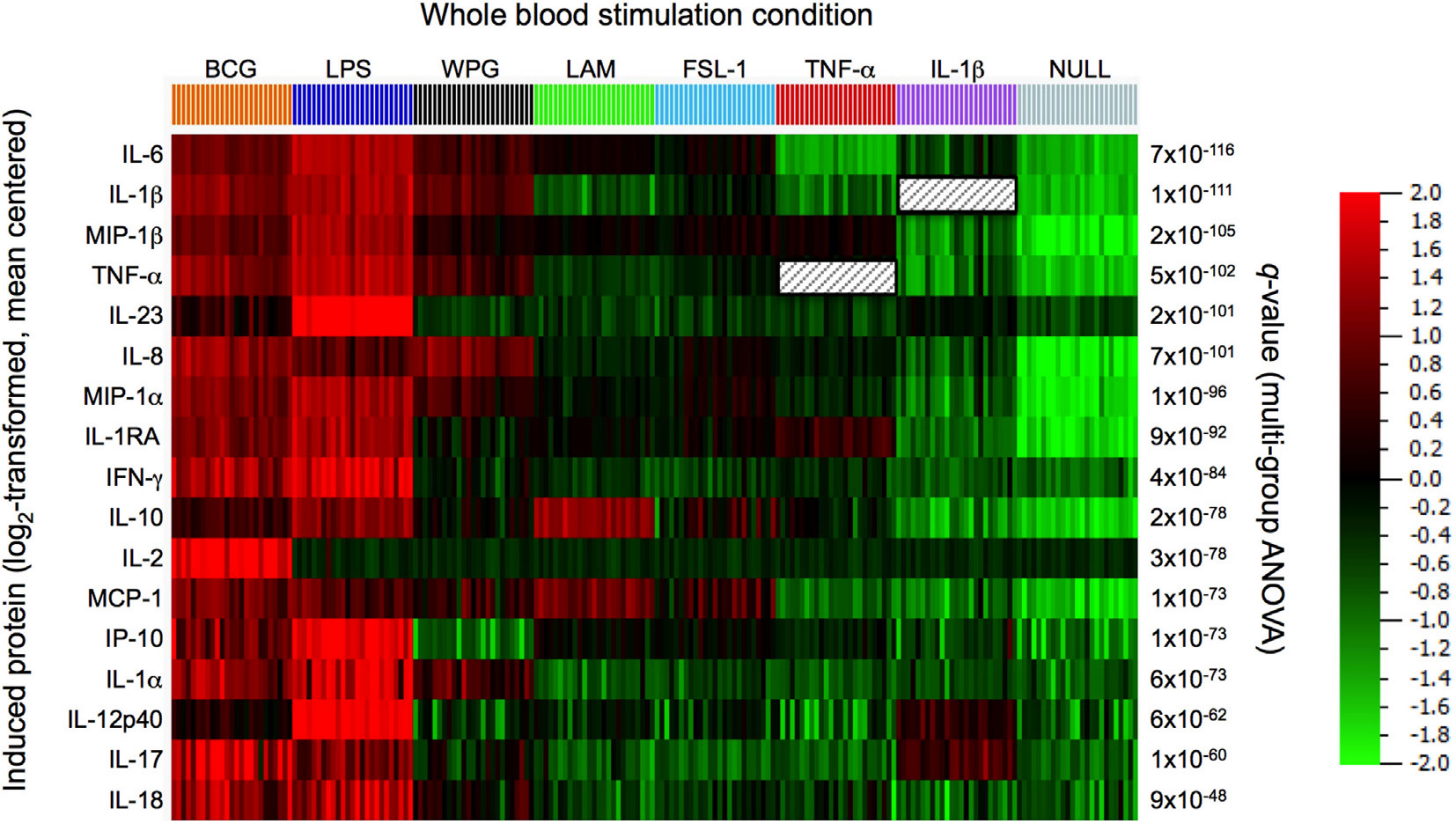
Comparison of protein responses induced by bacillus Calmette–Guérin (BCG) and its agonist ligand components. Clustering was performed on the data set obtained from 22 h whole-blood stimulation by BCG (3 × 10^5^ CFU), lipopolysacharride (LPS, 10 ng/ml), whole protein glucan (WPG, 40 µg/ml), lipoarabinomannan (LAM, 10 µg/ml), the synthetic lipoprotein Pam2C (FSL-1, 2 µg/ml), tumor necrosis factor-alpha (TNF-α, 10 ng/ml), interleukin 1-beta (IL-1β, ng/ml), and buffer control (null condition). A heat map is shown, samples ordered by stimulation and protein features hierarchically clustered using the 17 most differentially induced proteins as defined by ANOVA (filtered to include analytes with *q* value < 0.01).

**Figure 3:**
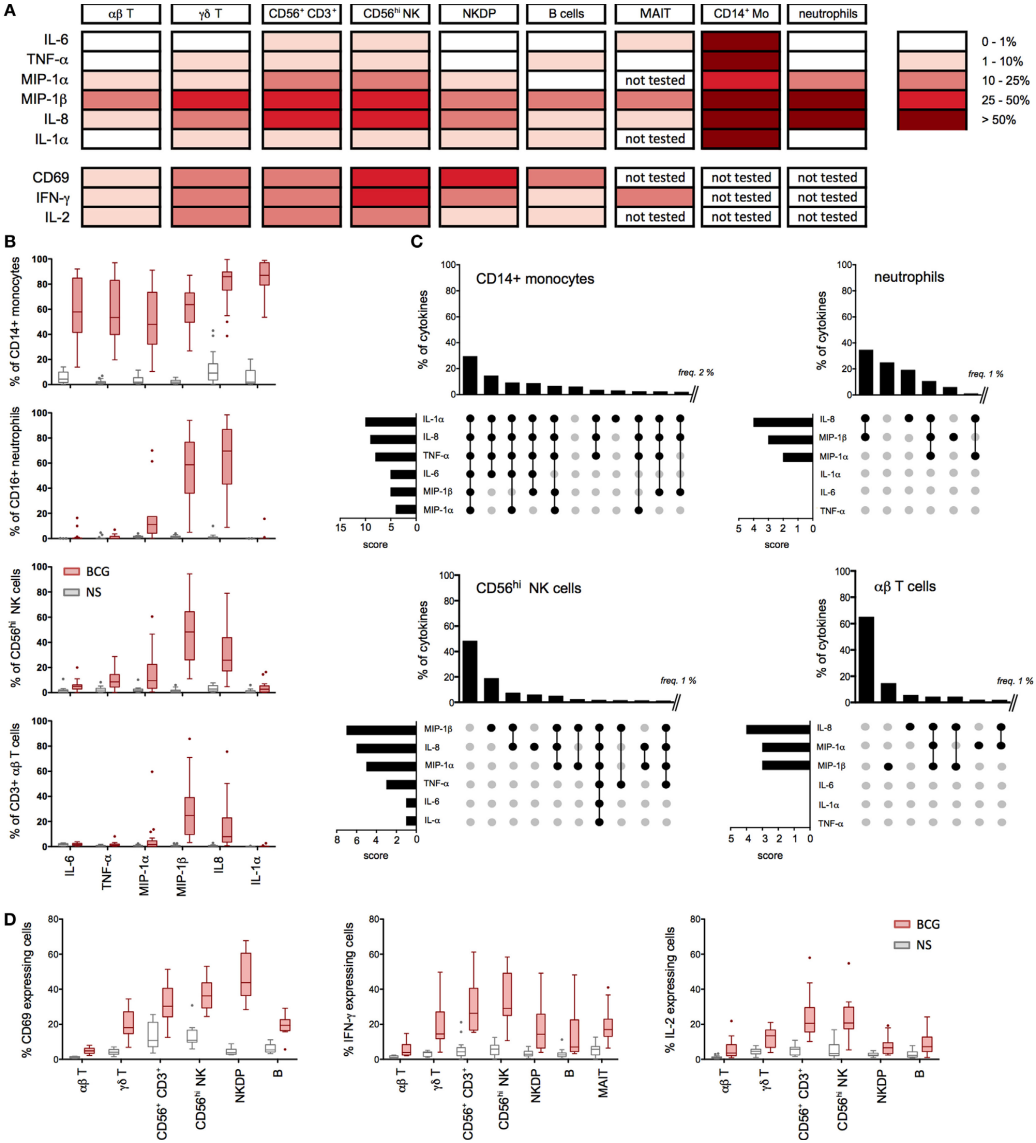
Multiple cell types contribute to the bacillus Calmette–Guérin (BCG) protein signature. **(A–D)** Whole-blood samples from 15 to 20 healthy donors were stimulated with BCG for 16 h in the presence of Brefeldin A (added 1 h after BCG) and assessed for cytokines production using specific cytometry panels (Table S2 in Supplementary Material). **(A)** Summary table representing the median values for the percentage of cytokine- and chemokine-positive cells (background signal from NS cells subtracted, *n* = 15–20) after 16 h of stimulation with BCG. **(B)** Tukey box-whisker plots indicate the percentages of cytokine-positive cells for CD14^+^ monocytes (*n* = 20), CD16^hi^ neutrophils (*n* = 18), CD56^hi^ NK cells (*n* = 20), and αβ T cells (*n* = 20). NS: non-stimulated condition. **(C)** Boolean gating analyses of the expressed cytokines measured in **(A)** are shown for monocytes, neutrophils, CD56^hi^ NK cells, and αβ T cells. Vertical bar graphs represent the percentage of each cytokines combination above 2% for monocytes and above 1% for other populations. Data are ranked from the greatest to the least expressed gated population of cytokine-producing cells. Horizontal bar graphs indicate the rank order of cytokine expression; the score represents the number of times each cytokine is present in a Boolean combination. The cytokines expressed in each combinatorial gate are represented with a black dot, connected by a solid black line. The cytokines not expressed are indicated by unconnected gray dots. **(D)** Tukey box-whisker plots indicate the expression of IFN-γ, IL-2, and CD69 for the indicated cell populations.

**Figure 4:**
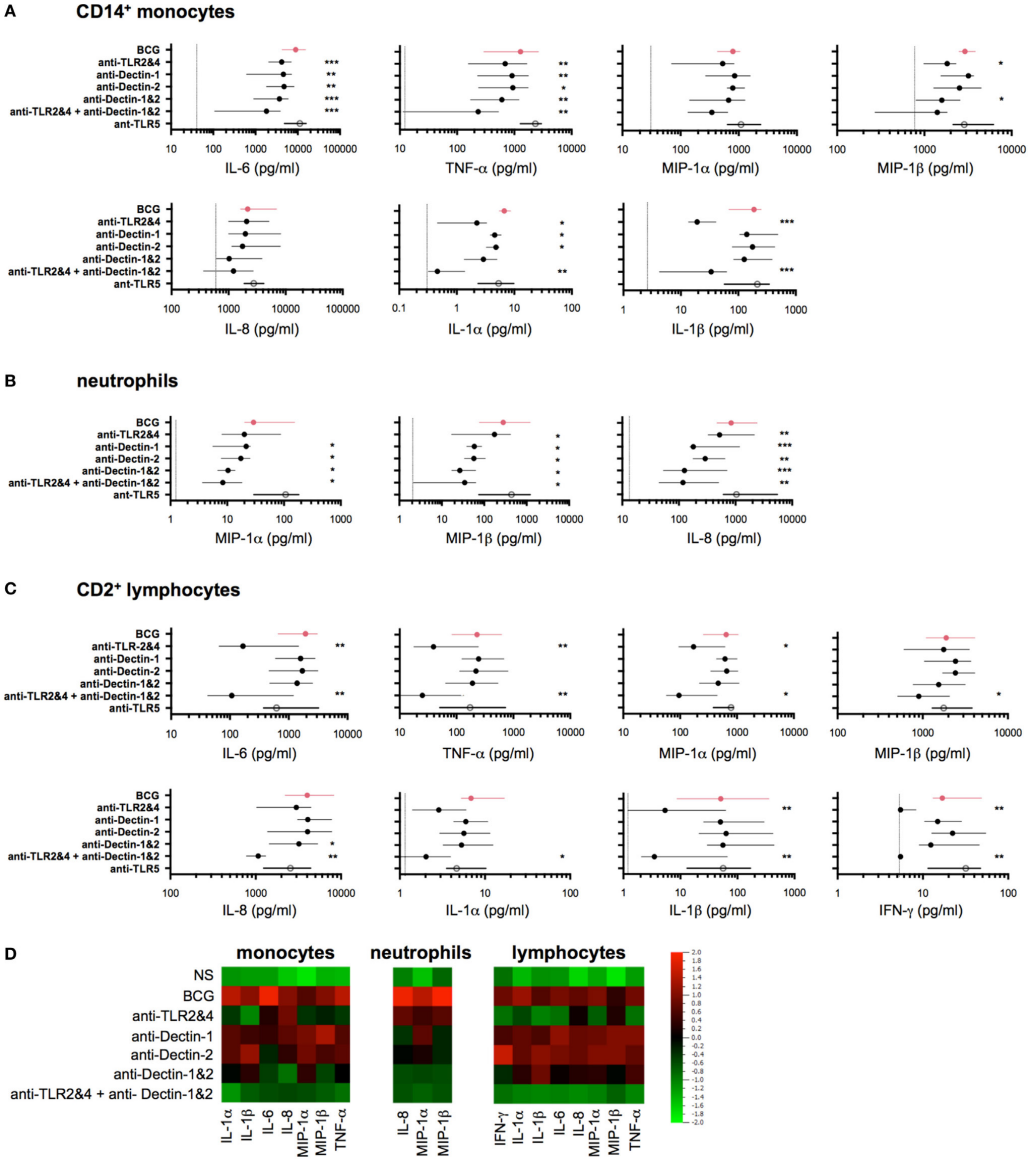
Bacillus Calmette–Guérin (BCG) engagement of multiple toll-like receptors (TLRs) and C-type lectin-like receptors accounts for early release of induced cytokines. **(A–C)** Concentrations of selected cytokines measured by Luminex assay from purified CD14^+^ monocytes [**(A)**, *n* = 5–8], CD66b^+^ neutrophils [**(B)**, *n* = 8)], and CD2^+^ lymphocytes [**(C)**, *n* = 8] each stimulated with BCG in the presence of indicated neutralizing antibodies (black circles) or isotype control antibodies (red circles) or neutralizing anti-TLR5 (white circle), for 5 h (monocytes and neutrophils) or 16 h (lymphocytes). Filled circles indicate the median concentration for each condition, and the lines represent the interquartile (IQR1/3) range. The dotted lines indicate the respective median values for unstimulated samples; if not shown, the null condition was below the limit of detection for the given assay. **(D)** Heat map representation using the median concentration determined from data represented above. *p* Values were determined by the paired Student’s *t* test and false discovery rate corrected for multiple analyte testing. *q ≤ 0.05; **q ≤ 0.01; ***q ≤ 0.001 as compared to BCG + isotype control antibody.

**Figure 5:**
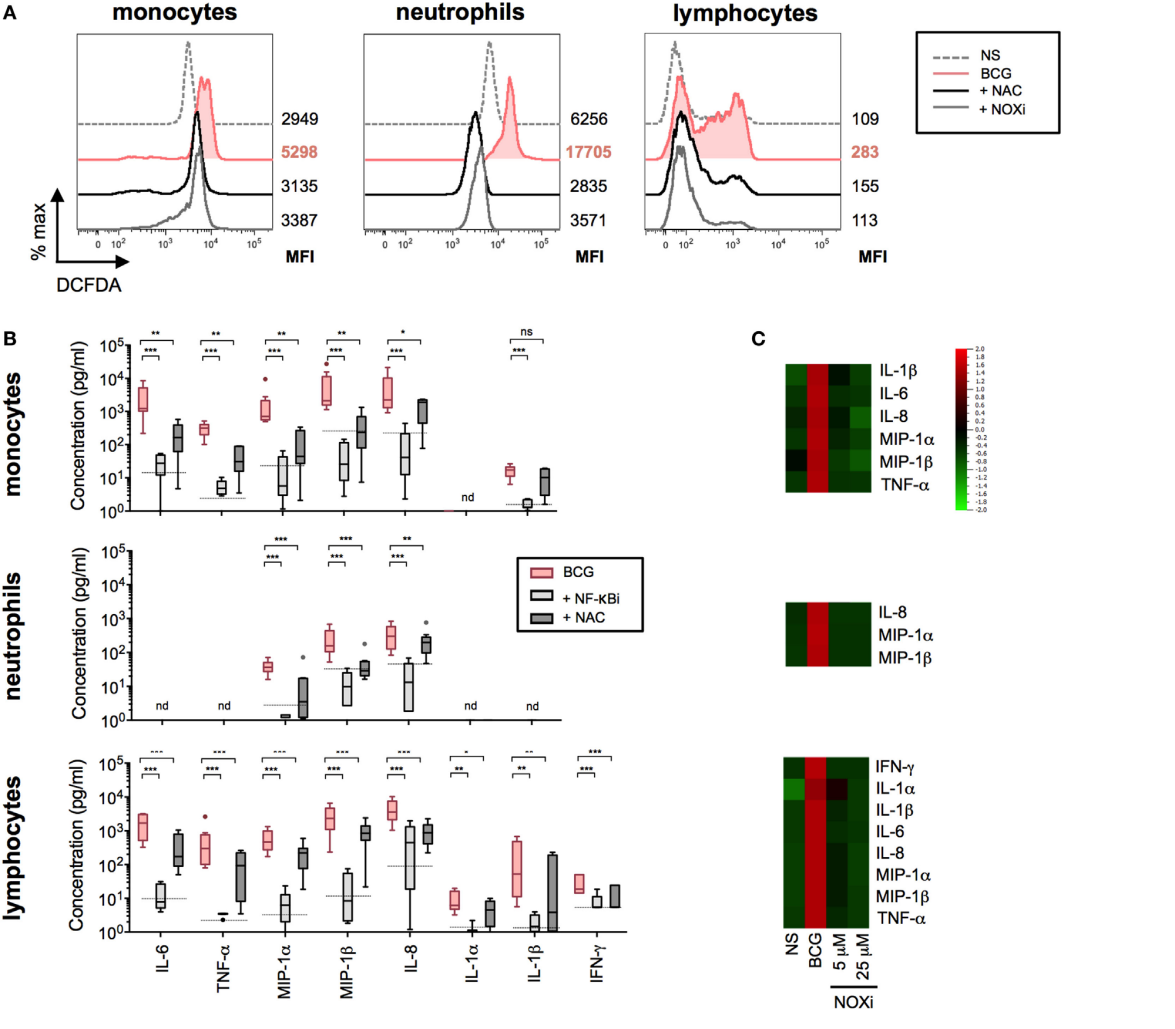

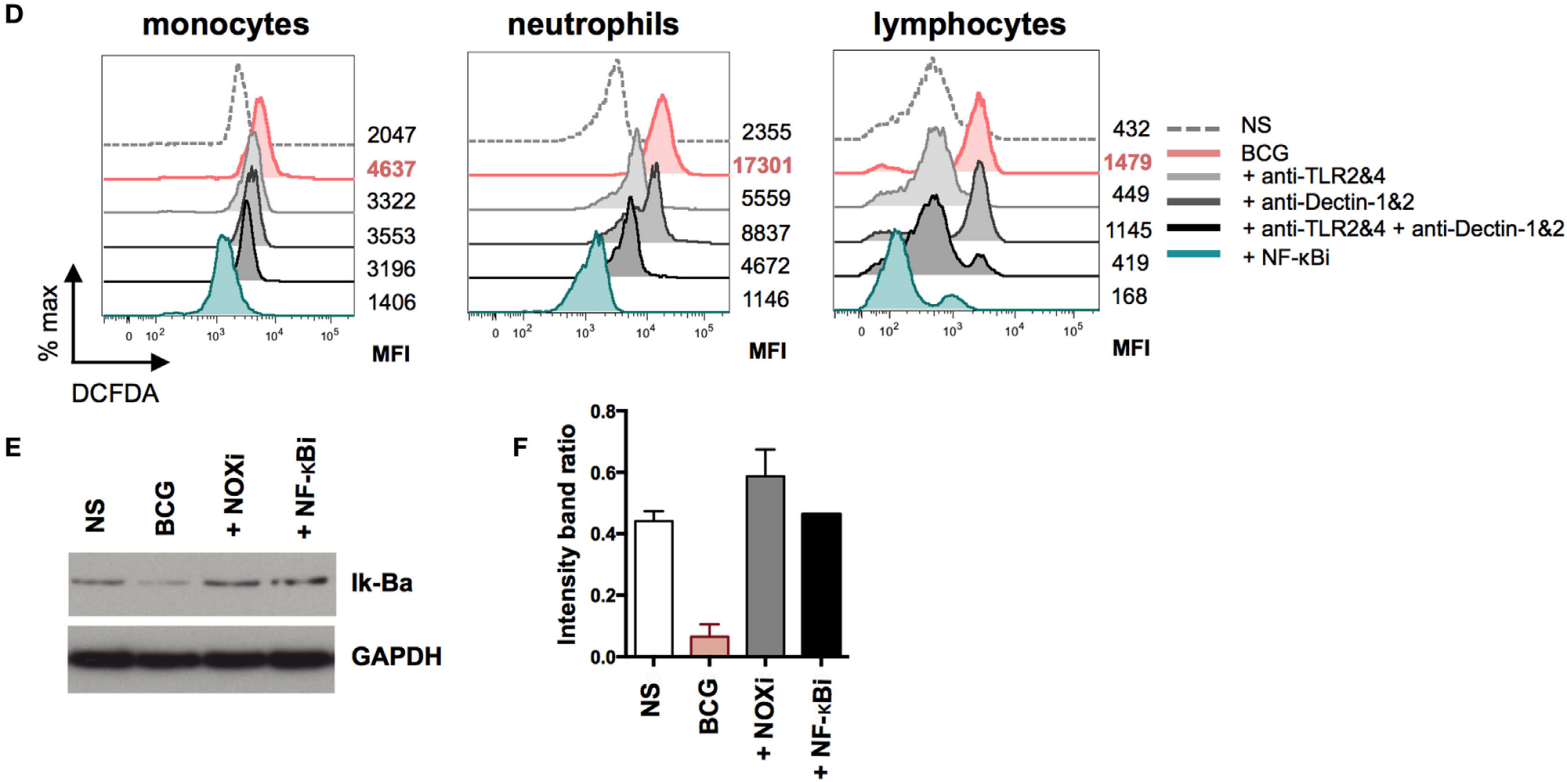
Bacillus Calmette–Guérin (BCG)-induced NF-κB and reactive oxygen species (ROS) activation results in the coordinated production of cytokines. **(A)** Representative histograms showing intracellular ROS production, measured by flow cytometry using dichlorofluorescin diacetate (DCFDA) assay on purified monocytes, neutrophils, and lymphocytes (*n* = 2) incubated with media control (dotted line); BCG in the presence of DMSO vehicle control, 5 mM *N*-acetyl-l-cysteine (NAC), or 5 µM of NOXi for a total of 5 h. **(B)** Concentrations of cytokines were determined by Luminex assay for purified monocytes (*n* = 9), neutrophils (*n* = 9), and lymphocytes (*n* = 9) stimulated with BCG in the presence of DMSO vehicle control, or NF-κB inhibitor, or NAC. The dotted lines indicate the median value for unstimulated samples. nd, not detected. *p* Values were determined by the paired Student’s *t* test and false discovery rate corrected for multiple analyte testing. *q ≤ 0.05; **q ≤ 0.01; ***q ≤ 0.001 as compared to BCG + DMSO control. **(C)** Heat map representation using median concentration measured of the listed cytokines for purified monocytes (*n* = 4), neutrophils (*n* = 4) and lymphocytes (*n* = 4) stimulated with media control [non-stimulated (NS)], BCG in the presence of vehicle control DMSO, or with 5 or 25 µM of NOXi, for 5 or 16 h for lymphocytes. **(D)** Representative histograms of intracellular ROS measured on purified monocytes (*n* = 6), neutrophils (*n* = 8), and lymphocytes (*n* = 6) stimulated for a total of 5 h with media control, BCG plus isotype control antibodies, BCG in the presence of neutralizing anti-TLR2&4, anti-Dectin-1&2, anti-TLR2&4 + anti-Dectin-1&2, or NF-κB inhibitor. The MFI values for each condition are shown to the right of the histograms. **(E)** Immunoblot analysis of Iκ-Bα and GAPDH from total leukocytes stimulated for 45 min with media alone (NS), BCG and DMSO vehicle control, BCG in the presence of NOX inhibitor, or NF-κB inhibitor (*n* = 3). **(F)** Band intensity was calculated using the ImageJ software, and the ratio of Iκ-Bα/GAPDH is represented by histograms.

**Figure 6:**
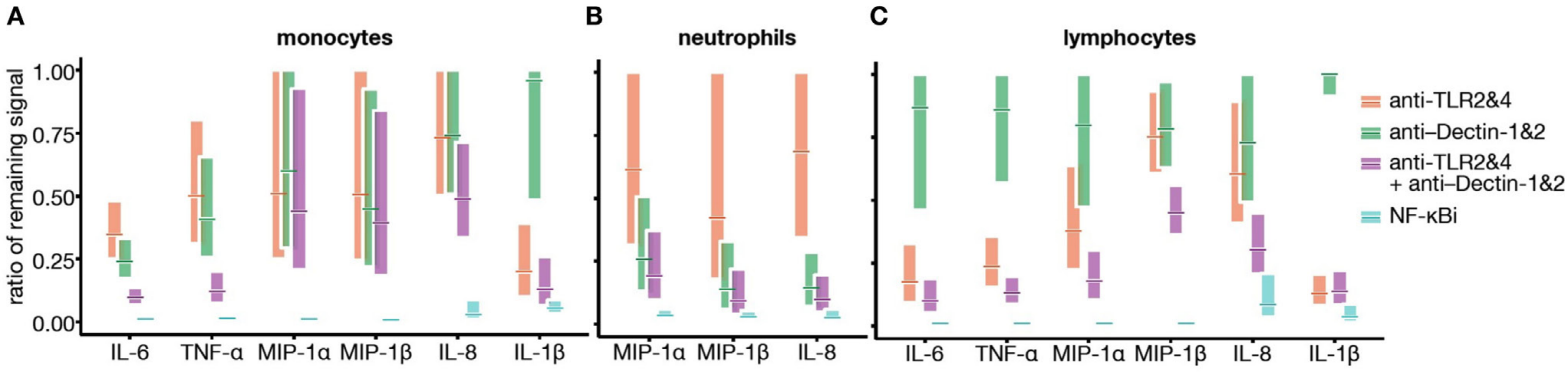
Toll-like receptor (TLR) and Dectin engagement by bacillus Calmette–Guérin (BCG) promotes NF-κB activation in an additive manner. **(A–C)** Data from Figures 4A–C and 5B were used to develop a mathematical model, querying whether TLR2&4 and Dectin-1&2 engagement is overlapping, additive, or synergistic in signaling *via* NF-κB. Cytokine concentrations were log-transformed, and ratio of remaining signal over BCG control condition was calculated for monocytes **(A)**, neutrophils **(B)**, and lymphocytes **(C)**.

**Figure 7:**
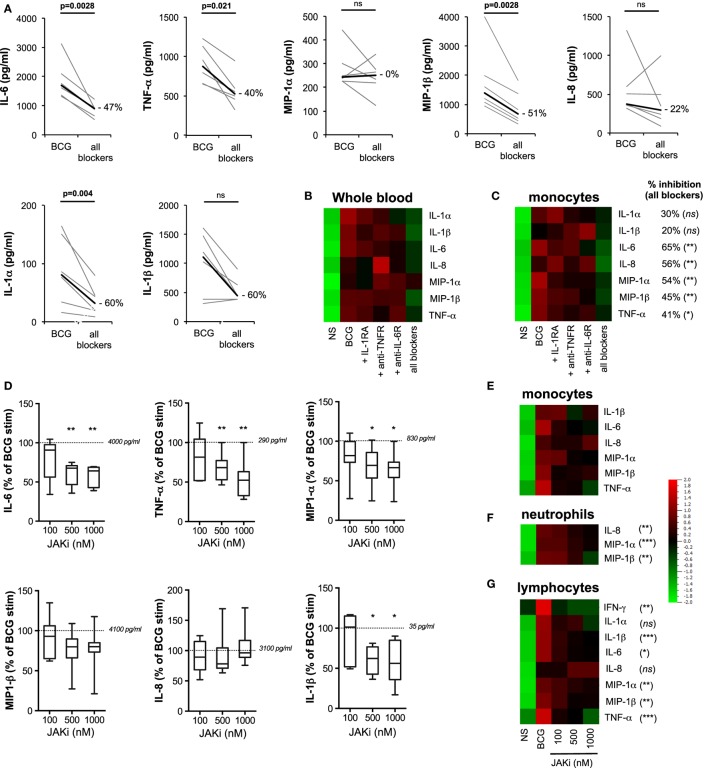
Feed-forward action of induced cytokines amplifies innate response to bacillus Calmette–Guérin (BCG). **(A–C)** The concentrations of cytokines were measured for whole blood and purified monocytes from healthy donors stimulated for 5 h with BCG in the presence of recombinant IL-1RA, anti-TNFR, and anti-IL-6R antibodies (referred to as “all blockers,” *n* = 6). **(A)** Detailed results from whole blood were represented as pair analyses, each line indicates a donor and the thick black line represents median values. Heat maps’ representation of the median concentration for the listed cytokines for whole blood [**(B)**, *n* = 6] and purified monocytes [**(C)**, *n* = 4–8], stimulated with BCG in the presence of the indicated blocking receptor reagent(s), BCG in the presence of isotype control antibodies, or buffer control [non-stimulated (NS)]. Percent inhibition between “all blockers” condition and BCG condition is indicated for each cytokine. **(D–G)** Purified monocytes (*n* = 7), neutrophils (*n* = 9), or lymphocytes (*n* = 7) were stimulated by BCG in the presence of increasing doses of the JAK1/2 inhibitor Ruxolitinib, and the quantification of cytokines were measured using Luminex assay. **(D)** Box-whisker plots indicate the percentage inhibition as compared to BCG stimulation for purified monocytes (normalized to 100% across experiments). The dotted lines indicate the median values for BCG stimulation for each cytokine. **(E)** Heat maps represent the median concentration determined for monocytes, **(F)** neutrophils, and **(G)** lymphocytes. *p* Values were determined by the paired Student’s *t*-test and false discovery rate corrected for multiple analyte testing. *q ≤ 0.05; **q ≤ 0.01; ***q ≤ 0.001 as compared to BCG + isotype control or DMSO control.

Data presented in Figures 1 and 2 were obtained using samples from the *Milieu Intérieur* Healthy Donor Cohort. Details are provided in Ref. (11) and information about the cohort was registered at http://www.clinical-trials.gov (identifier NCT01699893). Data presented in Figures 3–7 were obtained using fresh whole blood from healthy volunteers, collected in heparin coagulant tubes, and procured from Etablissement Français du Sang (EFS). The collection protocols were designed and conducted in accordance with the ethical principles of the Declaration of Helsinki and Good Clinical Practices as outlined in the ICH Guideline for Good Clinical Practices.

### TruCulture Whole-Blood Stimulation

TruCulture stimuli were resuspended in a volume of 2 ml buffered media and maintained at −20°C until the time of use. Blood was obtained from the antecubital vein by a 60 ml syringe containing sodium-heparin (50 IU/ml final concentration). Within 15 min of collection, 1 ml of whole blood was distributed into each of the prewarmed TruCulture tubes, inserted into a dry block incubator, and maintained at 37°C (±1°C) room air for 22 h. At the end of the incubation period, tubes were opened and a valve was inserted in order to separate the sedimented cells from the supernatants and to stop the stimulation reaction. Liquid supernatants were harvested, aliquoted, and immediately frozen at −80°C until time of use.

For the kinetic of cytokines release (Figures 1B–D), TruCulture tubes were maintained at 37°C (±1°C) for a 30 h time course, with the indicated time points: 0 (prior to the stimulation), 0.5, 1, 2, 3, 4, 6, 8, 10, 12, 16, 22, and 30 h. At the end of each incubation period, tubes were centrifuged at 500 rpm, 20°C, for 5 min and liquid supernatants were harvested, aliquoted, and immediately frozen at −80°C until the time of use. All the samples described earlier were then analyzed with Luminex xMAP technology.

### BCG Preparation

*Mycobacterium bovis* BCG Connaught strain was grown in 7H9 Middlebrook culture medium, supplemented with 0.4% glycerol (Sigma, USA) and 10% ADC enrichment (Becton Dickinson, France). After 2–3 weeks of growth, an aliquot of the culture was harvested and washed in 1× Dulbecco’s phosphate-buffered saline (DPBS; Gibco, France) for 8 min at 4,000 rpm. The bacterial pellet was resuspended in 5 ml of DPBS and then transferred to a GentleMACS M tube (Miltenyi Biotec, France). The RNA_01.01 protocol of the GentleMACS Dissociator (Miltenyi Biotec) was used to disrupt aggregated bacteria. The optical density of the bacteria was read at 600 nm (1 OD = 1 × 10^8^ CFU/ml), and bacteria was adjusted to the predetermined concentration and immediately used.

### Intracellular Cytokines Staining

Fresh heparinized whole blood from 15 to 20 healthy donors from EFS was stimulated with 10^6^ CFU/ml of BCG in 5 ml polypropylene capped tubes in the presence of RPMI-1640 GlutaMAX (half dilution; Gibco) at 37°C for 16 h. GolgiPlug (BD Biosciences, France) was added to each tube 1 h after BCG. At the end of the stimulation period, 2 mM of EDTA (Life Technologies, France) was added for 10 min at 37°C to detach adherent cells and the samples were incubated with antibodies described in Table S2 in Supplementary Material for 15 min at room temperature (RT), protected from light. The red blood cells were lysed and fixed using BD lysis buffer, centrifuged at 300 × *g* for 5 min, and incubated with 1 ml of BD Perm/wash buffer for 5 min at 4°C. The cells were washed and stained with fluorescent antibodies specific to cytokines (listed in Table S2 in Supplementary Material) in a total volume of 100 µl, at 4°C for 1 h. Stained cells were washed in DPBS and acquired on a BD LSR Fortessa (BD Biosciences).

### Isolation of CD66^+^ Neutrophils and CD14^+^ and CD2^+^ Cells

Fresh heparinized whole blood from healthy donors was utilized for neutrophil, CD14, and CD2 purifications. Whole blood was lysed (155 mM NH_4_Cl, 10 mM KHCO_3_, and 0.1 mM EDTA in distilled water) for 10 min at RT and washed twice for 10 min at 300 × *g*. CD66b^+^ neutrophils were isolated by positive selection using the CD66abce Microbead kit (Miltenyi Biotec) according to manufacturer’s protocol. Purity was assessed by flow cytometry using BV421-conjugated anti-CD66b and APC-conjugated anti-CD14 (BD Biosciences). The purity of the enriched population was >98%. CD14^+^ and CD2^+^ cells were positively isolated from the PBMC fraction obtained by Ficoll-Hypaque Plus gradient separation (GE Healthcare, France), using CD14 and CD2 Microbeads (Miltenyi Biotec), according to manufacturer’s protocol. Briefly, CD14^+^-labeled cells were first enriched on positive selection columns and the depleted fraction containing total lymphocytes was washed and stained with CD2 Microbeads and isolated on positive selection columns. Purity of CD14 and CD2 enriched fractions was assessed by flow cytometry using APC-conjugated anti-CD14 (BD Biosciences) and efluor450-conjugated anti-CD3 (Ebioscience, France) and was >98%. Viability of the samples following each purification procedure was assessed by Trypan blue staining and was >97% for all experiments.

### Blocking Reagents

All neutralizing antibodies or chemical inhibitors were first tested in a dose-dependent manner using adequate controls to optimize conditions. Blocking reagents were added to whole blood or purified cell populations 1 h prior to the stimulation. We utilized the following blockers: anti-hTLR2 and -TLR4 (pab-hstlr2/4, 25 µg/ml), anti-hTLR5 (maba2-htlr5, 0.5 µg/ml), anti-hDectin-1 and -2 (mabg-hdect/-hdetc2, 10 µg/ml), and Ruxolitnib (tlrl-rux, 0.1–1 µM) from InvivoGen (France); anti-hCD120a (clone MABTNFR1-B1, 5 µg/ml) and anti-hCD130 (clone AM64, 5 µg/ml) from BD Biosciences; IL-1RA (Kineret^®^100mg, 400 µg/ml) and *N*-acetyl-l-Cystein (NAC, 5 mM) from Calbiochem (Merck Millipore, France); and NADPH oxidase inhibitor (VAS2870, 5–25 μM) and NF-κB inhibitor (BAY 11-7085, 1 μM) from Sigma.

### Stimulation of Whole-Blood Samples and Isolated Cells in the Presence of Blocking Reagents

Diluted whole blood (v/v in RPMI-1640 GlutaMAX, 200 µl final), or 50,000 enriched CD66^+^ and CD14^+^ cells, or 10,000 enriched CD2^+^ cells were incubated with the blockers previously described or corresponding vehicle controls (DMSO or isotypes control) in RPMI-1640 GlutaMAX supplemented with 10% of filtered fetal calf serum (FCS, Gibco) in 96-round well plates, for a total period of 5 or 16 h for CD2^+^ cells, at 37°C. After 1 h of incubation, 10^6^ CFU/ml of BCG (final concentration) was added to each well (with the exception of one well for the unstimulated condition), and samples were incubated for the rest of the indicated time. At the end of the incubation period, supernatants were harvested and stored at −20°C until the time of use. Viability of cells was assessed by Trypan blue counting and was >90%.

### Multianalyte Profiling Identification of Inflammatory Signatures

Supernatants from whole-blood stimulation systems (*Milieu Intérieur* healthy donors, Figures 1 and 2) were analyzed with Luminex xMAP technology. Samples were measured according to CLIA guidelines (Myraid RBM, USA). The 32 measured proteins were organized on three multiplex arrays, and a single batch of reagents was used for testing all samples (Table S1 in Supplementary Material). Supernatants from stimulated whole blood or purified cells (EFS healthy donors, Figures 3–6) were analyzed using combined ProcartaPlex^®^ Simplex Immunoassays (Ebioscience) for IL-6, TNF-α, MIP-1-α, MIP-1β, IL-8, IL-1α, and IL-1β plus IFN-γ (for lymphocyte supernatants only). Assays were run according to the manufacturer’s protocol. Data were collected using the MAGPIX^®^ technology or Luminex^®^ 100 System.

### Measurement of Total Cellular ROS

Intracellular accumulation of ROS was monitored by flow cytometry using the cell permeant reagent 2′,7′-dichlorofluorescin diacetate (DCFDA), a fluorogenic probe that measures ROS activity within the cell (Abcam). Monocyte, neutrophil, and lymphocyte populations were isolated as previously described and incubated with media alone (RPMI-1640 GlutaMAX) supplemented with 10% of FCS, or with 10^6^ CFU/ml of BCG in the presence or absence of selected blockers (added 1 h before BCG), for a total period of 5 h at 37°C. Cells were centrifuged in DPBS at 300 × *g* for 5 min and incubated with the DCFDA solution (20 µM) for 30 min at 37°C, protected from light. Samples were then immediately run on an FACS LSR Fortessa using the 488 nm laser.

### Western Blot Analyses

Total leukocytes obtained after lysis of fresh whole blood were stimulated with media alone (unstimulated condition), or with 10^6^ CFU/ml of BCG and vehicle control DMSO, or in the presence of 5 µM of NOX inhibitor (VAS2870) or 1 µM of NF-κB inhibitor, for 30 and 45 min. Cell pellets were washed twice in cold DPBS and stored at −20°C until use. Samples were lysed in DPBS containing 1% Non-idet P 40 substitute (NP-40; Sigma-Aldrich) supplemented with protease and phosphatase inhibitors (Roche). Total proteins were measured using bicinchoninic acid assay (BCA; ThermoFisher), and 30 µg of proteins were loaded on a 4–12% BIS-TRIS gel (Biorad). Proteins were transferred to PVDF membrane and blotted with anti-IκBα (rabbit polyclonal, #9242; Cell Signaling) or anti-GAPDH (rabbit monoclonal, #2118; Cell Signaling) in Tris-buffered saline containing 0.05% of Tween 20 and 5% of bovine serum albumin. Secondary rabbit HRP-coupled antibodies were detected using the SuperSignal West Pico Chemiluminescent Substrate (ThermoFischer).

### Flow Cytometry Analyses

Flow cytometry data were analyzed using FlowJo v9.5. Cell doublets were excluded using side scatter-area versus forward scatter-width and side scatter-width versus forward scatter-area parameters. Lymphocyte populations analyzed in panel #1 were selected from a [CD3^+^ and CD3^−^]/CD14^−^ gate to exclude any contamination of monocytes. MAIT cells were gated first on CD3^+^ cells, and the gating strategy described in Figure S1 in Supplementary Material was then applied. Cytokine co-expression from the different cell populations was assessed by Boolean gating on FlowJo. Background non-stimulated values were first subtracted for all combinations, and Boolean gate analysis performed on the six proteins from panel #1.

### Linear Modeling of Inhibition Data

Cytokine concentrations were imported in R (CRAN v. 3.2.3) and log10-transformed. For each analyte, we used a linear regression to model the log-transformed protein level *y*_d,i_ of donor d with inhibitor i:

$$y_{\text{d,i}}=\alpha +\alpha_{\text{d}}-\beta_{\text{i}}+\varepsilon_{\text{d,i}}$$

where α is fixed, α_d_ has a different value for each donor, and β_i_ has different values for each inhibitor; the ε_d,i_ are independent identically distributed normal random variables. Estimates for the ratio of remaining signal was given by 10^β^, and its 95% confidence interval by [10^β−1.96 SE(β)^, 10^β+1.96 SE(β)^], where β and se(β) denote the estimate and SE of β, respectively. To test for evidence of a synergistic effect of TLR and Dectin inhibitors, we fitted a slightly different model, namely:

$$y_{\text{d,i}}=\alpha +\alpha_{\text{d}}-\delta_{\text{i,TLR}}\beta_{\text{TLR}}-\delta_{\text{i,Dectin}}\beta_{\text{Dectin}}-\delta_{\text{i,T}+\text{D}}\beta_{\text{T}+\text{D}}+\varepsilon_{\text{d,i}}$$

where α is fixed, α_d_ has a different value for each donor, δ_i,TLR_ has value 1 if and only if i = iTLR or iTLR + iDectin; δ_i,Dectin_ has value 1 if i = iDectin or iTLR + iDectin, 0 otherwise; δ_i,T+D_ has value 1 if i = iTLR + iDectin, 0 otherwise; the ε_d,i_ are independent identically distributed normal random variables. We then tested the hypothesis H_0_: β_T+D_ = 0 for each analyte/population pair.

### Statistical Analyses

Principal component analysis was performed with Qlucore Omics Explorer, v.23 (Qlucore). For statistical analyses, as protein data are generally close to a log-normal distribution (12), data were log-transformed using Prism 6 GraphPad software and a paired Student’s *t*-test, which is shown to be extremely robust against non-normality (13), was performed. To account for multiple testing, *p*-values in each condition were corrected using false discovery rate (FDR), called *q* values. *q* Values ≤ 0.05 were considered significant (ns, not significant; *q ≤ 0.05; **q ≤ 0.01; ***q ≤ 0.001).

## Results

### BCG Induces a Rapid and Robust Protein Signature

We initiated our study by establishing a BCG-induced protein signature. Samples were collected in the context of the *Milieu Intérieur* project, using syringe-based medical devices that contained 3 × 10^5^ live BCG (Connaught strain). After 22 h stimulation, host proteins were quantified using the *x*MAP Luminex technology. Seventeen of the 32 proteins tested (see Table S1 in Supplementary Material) showed a >2 median fold change across 25 healthy donors [reported recently in Ref. (6)] and statistical analysis comparing BCG stimulation to the null condition showed an FDR corrected *q*-value < 0.01 for these 17 proteins (Figure 1A). The observed BCG protein signature reflected the activation of both innate immune cells and lymphocytes, as indicated by the production of IL-6 and IL-2, respectively.

We next investigated the sequential secretion of these proteins, measured after whole-blood stimulation of five healthy donors, comparing BCG and null conditions during a 30 h time course (Figures 1B–D). Strikingly, we observed four discrete groupings of induced cytokines and chemokines with representative data showing: (i) immediate early secretion of TNF-α, IL-8 (CXCL8), MIP-1α (CCL3), and MIP-1β (CCL4) was evident between 30 and 60 min following stimulation; (ii) early release of IL-6, IL-1β, and IL-1RA occurred within 3 h; (iii) intermediate secretion of IL-1α, IL-10, IL-12p40, and MCP-1 (CCL2) was observed between 8 and 10 h; and (iv) late secretion of IP-10, IL-17, IL-18, IL-23, IFN-γ, and IL-2 could be detected 16 h post-BCG stimulation (Figures 1B,C). With the exception of IFN-γ and IL-2, all molecules reached a plateau by 20 h. Together, these data revealed a complex response to BCG stimulation that begins as early as 30 min post-engagement, schematized based on the kinetics of secretion (Figure 1D). This complex response likely reflects the engagement of multiple host sensors acting on diverse cell types. Moreover, the rapid induction of host cytokines suggested that feed-forward activation of cytokine receptor signaling pathways may contribute to the observed BCG molecular signature.

To examine how BCG, as a complex stimulus, compares to derivative activation of defined host sensors/receptors, we evaluated the induced host cytokine and chemokine response to key TLR and CLR sensors. Specifically, we tested LAM (component of the mycobacterial cell wall and ligand for TLR2), whole protein glucan (ligand for Dectin-1), FSL-1 (synthetic diacylated lipoprotein; ligand for TLR 2/6); ultrapure lipopolysacharride (LPS)-EB (ligand for TLR4); as well as the engagement of TNF receptor and IL-1R, with the use of TNF-α or interleukin-1-beta (IL-1β), respectively, comparing the results to BCG whole-blood stimulation. LAM and LPS engagements of TLR2 or TLR4, respectively, were validated using blocking antibodies (Figure S2 in Supplementary Material). Protein expression data for each of the 17 analytes were centered to a mean value of zero and scaled to unit variance to compare the results across the various stimulation conditions. Results were stratified based on the stimulus, and differential expression of induced proteins was analyzed using ANOVA, with protein response data subjected to hierarchical clustering and presented using a heat map (Figure 2). We observed strong stimuli-specific clusters with a high degree of overlap between the BCG- and TLR4-induced protein responses. Of note, BCG was unique in its induction of IL-2, possibly a reflection of adaptive cell stimulation. Dectin-1 activation also triggered a broad cytokine response; as compared to TLR2 activation by LAM or FSL-1, which induced a narrower response. In all instances, however, the component ligands failed to account for the breadth of the BCG signature. These results led us to conclude that the BCG-induced response is more complex than the combined individual responses provoked by the component agonistic ligands.

### The BCG-Induced Protein Signature Is an Aggregate of Monocyte, Neutrophil, and Lymphocyte Activation

Given the broad expression of TLR, CLR, and cytokine receptors on leukocyte populations, we established single cell-based assays to assign the cellular source(s) of selected cytokines. While it is well established that BCG induces inflammatory cytokines, prior studies have not mapped the expression to defined leukocytes cell types. We utilized multiparametric flow cytometry, designing three immunostaining panels to classify nine distinct cell populations (Figure S1 and Table S2 in Supplementary Material), while simultaneously evaluating intracellular protein expression. We selected eight different proteins from the 17 that have been identified in BCG-stimulated whole-blood samples (Figure 1A). In addition, these proteins cover the four different kinetic stages of the BCG response signature represented in Figures 1B–D and prior data support their role in the context of human mycobacterial disease(s) and/or vaccination by BCG (14, 15). Representative examples from single donors illustrate the induction of cytokines and chemokines by CD14^+^ monocytes, CD16^hi^ neutrophils, and CD56^hi^ NK cells, respectively (Figure S1 in Supplementary Material). In addition, we evaluated the activation of lymphocyte populations and measured by increased expression of CD69 and the production of IFN-γ and/or IL-2 (Figure S1 in Supplementary Material).

Median values for the percentage of cytokine and chemokine positive cells were determined for greater than 15 donors. Aggregated results show distinct protein signature for each selected cell types. Specifically, the data highlight a dominant role for monocytes, which highly expressed all selected cytokines (Figures 3A,B). While the role of monocytes confirmed results from prior studies (16, 17), the protein signature of NKT-like and CD56^hi^ NK populations was diverse and distinct from other lymphocyte populations tested (Figure 3A). For NKT-like cells, the broad response may be due to engagement of CD1-restricted presentation of BCG lipids (18). Additionally, we observed a unique pattern for CD16^hi^ neutrophils with protein expression restricted to MIP-1α, MIP-1β, and IL-8. Interestingly, we detected MIP-1β and IL-8 expressions on all the leukocyte cell types investigated (Figures 3A,B). Using Boolean gating analysis, we evaluated the 64 different combinations (2^6^, for the six innate cytokines and chemokines measured), defining the respective percentage of positive cells (Figure 3C). As shown, ~30% of monocytes produced all six proteins and <10% expressed none of the six measured cytokines and chemokines. By comparison, neutrophils produced only 1–3 protein analytes per cell, yet were similarly responsive with ~75% making at least one chemokine. While a lower proportion of CD56^hi^ NK and αβ T cells responded to BCG (e.g., 50% and 60% of cells making no measured cytokine, respectively), we nonetheless observed small numbers of CD56^hi^ NK cells making the full repertoire of proteins.

Following from the expression of inflammatory cytokines by lymphocytes, we further tested the upregulation of activation markers and the production of IFN-γ and IL-2. Strikingly, 25–60% of NK and CD56^+^ CD3^+^ NKT-like cells showed upregulation of the activation marker CD69 (Figure 3D). We also observed BCG stimulation of MAIT cells, showing that ~20% of cells respond by producing IFN-γ (Figure 3D). Additionally, a smaller proportion of αβ T cells and B cells responded to BCG. These data provide a detailed picture of the BCG-mediated inflammatory signature of leukocytes and reveal differences in their activation state, which helps to define the underlying variable host response to BCG. These results also confirmed our initial hypothesis that the BCG-induced protein signature represents a composite response involving diverse cell types.

### The First Wave of Induced Proteins Is Triggered by BCG Engagement of TLR2/4 and Dectin-1/2 Acting *via* NF-κB and ROS

We next hypothesized that the observed BCG-induced cytokines were the result of engaging multiple host sensors on three classes of cells: CD14^+^ monocytes, neutrophils, and CD2^+^ lymphocytes (which include NK and T cells). The initial response to BCG has been shown to act *via* the engagement of pattern recognition receptors (PRRs), or microbial sensors, expressed on immune cells (19). Thus, we investigated the role of two major TLRs for BCG (i.e., TLR2 and TLR4), and the C-type lectin receptors Dectin-1 and Dectin-2, which have been reported to interact with a wide variety of microbes, including mycobacteria (10, 20), yet their requirement on human cells remain to be elucidated. To assess the role of these host receptors, we utilized neutralizing antibodies specific for TLR2, TLR4, Dectin-1, and Dectin-2. Based on initial results that showed highly variable responses when using single blocking reagents, we focused on the combined neutralization of TLR2 and TLR4. Dectin-1 and Dectin-2 antibodies were tested singly or in combination (referred to as Dectin-1&2). To evaluate the combined effect of blocking all four receptors, we combined the four antibodies together (referred to as TLR2&4 + Dectin-1&2). As a response-based read-out, we measured the concentration of selected cytokines and chemokines (Figure 3). As a control, we utilized a neutralizing TLR5 antibody, which is not expected to inhibit BCG responses due to its lack of a flagellum, the agonist for TLR5 (21). In all cases, dose titration studies were performed using synthetic agonists to optimize conditions and demonstrate specificity of the reagents (Figure S2 in Supplementary Material).

For an overall view on the role of host sensors, we first conducted studies in whole-blood samples from healthy donors. The combined use of TLR2&4 neutralizing antibodies showed decreased IL-6, TNF-α, IL-1α, and IL-1β productions, whereas the expression of MIP-1α, MIP-1β, and IL-8 was highly variable and overall, only modestly impacted (Figure S3 in Supplementary Material). To better define the action of host sensors, we next utilized purified cell populations, isolated using a magnetic bead-based approach (achieving >98% of purity in all experiments). Purified populations were stimulated using 10^6^ CFU/ml of live BCG, in the presence of different neutralizing antibodies, and culture supernatants were harvested after 5 h for CD14^+^ monocytes and neutrophils, or 16 h for CD2^+^ lymphocytes. Notably, the control stimulation conditions verified that each cell type showed comparable protein stimulation profiles to those defined by flow cytometry (Figure 3A), with the exception of IL-1α, which is known to be an intracrine and is secreted at only low levels (22). Interestingly, the stimulation of purified lymphocytes supports their ability to directly respond to BCG, independently of myeloid cells.

The data obtained in the presence of the selected neutralizing antibodies revealed three distinct patterns of inhibition. For CD14^+^ monocytes, we observed a significant but modest decrease in cytokine production when using either TLR2&4 or Dectin-1&2 neutralization antibodies as compared to isotype control antibody treatment (40–60% inhibition for MIP-1β, TNF-α and IL-6, *q* < 0.05, Figure 4A). The combination of all blocking antibodies further decreased the production of IL-6, TNF-α, MIP-1α, and MIP-1β (while not significant for MIP-1α, Figure 4A). By contrast, IL-8 production was weakly affected by anti-TLR2&4, and IL-6 and TNF-α showed inhibition in the presence of the single Dectin blockers as compared to the other cytokines measured. We also observed a unique pattern of IL-1α and IL-1β expressions, which were both modulated by TLR2&4 blocking, with >80% reduction in signal as compared to control treatment (*q* < 0.05, Figure 4A). For neutrophils, we observed a distinctive activation profile in the presence of the selected blockers, with a considerable decrease of all three induced proteins when Dectin-1 or Dectin-2 signaling was inhibited. Despite expression of TLR2&4, the inhibitory effect by Dectin-1&2 was more pronounced. Nonetheless, inhibition of cytokine production was further enhanced by the combination of both pathways, showing ~80% of inhibition as compared to control (*q* < 0.05, Figure 4B). By contrast, results from lymphocytes supported a primary involvement of TLR2&4, since blocking of Dectin-1&2 showed little additional inhibitory effects (Figure 4C). Again, lymphocytes are known to express Dectin receptors; however, blockade revealed selective use of one pathway over the other (23, 24; and data not shown). To compare results across the different cell types, data were aggregated, log-transformed, and mean centered; a heat map representation is shown for the induced cytokines and chemokines (Figure 4D). These data confirmed the role of TLR2 and TLR4 in BCG-induced inflammatory cytokines and provided new evidence for both Dectin-1 and Dectin-2 in the initiation of host responses, Moreover, we established that despite broad expression of these host receptors on responding leukocytes (Figure S4 and Table S2 in Supplementary Material), BCG differentially engages PRRs on the three cell types studied.

We next investigated the intracellular pathways that are engaged as a result of host sensor engagement. It is well described that both ROS production and NF-κB activation are rapidly triggered following TLR and CLR binding. We therefore investigated the role of these signaling pathways in BCG-induced cytokine and chemokine productions on the three cell types. BCG-induced ROS was observed after 4 h stimulation using a cell-based reporter assay, validated by the use of the ROS scavenger NAC (Figure 5A). These data indicated that ROS was robustly produced in all cell populations when exposed to BCG as compared to media control conditions. Using an NF-κB-specific inhibitor (NF-κBi) and NAC, we next investigated the role of these two pathways on BCG-induced cytokine production. For all three purified cell populations, we observed a profound reduction of cytokine production when either NF-κB activation or ROS production was inhibited (Figure 5B). To establish the source of ROS, we tested an inhibitor selective for NADPH oxidase (VAS2870, referred to as NOXi). Indeed, NOXi treatment abrogated BCG-induced ROS (Figure 5A) and diminished cytokine/chemokine production in a dose-dependent manner (Figure 5C). Notably, we also excluded a role for BCG-induced mitochondrial ROS production using a MitoSox reporter assay (Figure S5 in Supplementary Material). These results indicate that BCG-induced cytokine production *via* the engagement of NF-κB and NOX-dependent ROS pathways in monocytes, neutrophils, and lymphocyte subsets.

To ascertain the connection between these two pathways, we tested whether PRR activation of NF-κB was required for the observed BCG-induced respiratory burst. We measured the production of ROS in purified cell populations after stimulation with BCG, in the presence or absence of NF-κBi, comparing it to the use of neutralizing antibodies specific for TLR2&4 and/or Dectin-1&2. Strikingly, we observed a dramatic reduction in ROS production when NF-κB activation was inhibited (Figure 5D; Figure S6 in Supplementary Material). In addition, we observed that neutralization of TLR2&4 or Dectin-1&2 decreased ROS in both monocytes and neutrophils, with an even stronger inhibition when using combinations of TLRs and CLRs blocking antibodies (Figure 5D; Figure S6 in Supplementary Material). For lymphocyte subsets, ROS production was exclusively inhibited by TLR2&4 blocking antibodies, again illustrating a critical role for TLR engagement as the mechanism of BCG activation in this cellular population. While these data indicate that NF-κB activation results in ROS production, it has been previously reported that ROS can act upstream of NF-κB activation (25, 26). We therefore tested the level of Iκ-Ba degradation during BCG stimulation, in the presence or absence of NOXi. As expected, NS cells showed an intact band for Iκ-Ba, and upon BCG exposure Iκ-Ba degradation was observed, a hallmark of NF-κB activation (Figures 5E,F). Strikingly, Iκ-Ba degradation was inhibited by the presence of NOXi, thus demonstrating a role for ROS as an upstream signaling molecule responsible for BCG-induced NF-κB activation. Similar results were obtained with NAC (data not depicted). These findings demonstrated that PRR engagement by BCG resulted in the activation of NF-κB and the generation of ROS, which intersected as co-dependent intracellular pathways responsible for triggering the production of inflammatory cytokines and chemokines.

### TLR2/4 and Dectin-1/2 Engagement Provide Additive Activation Signals and Their Relative Contribution Is Cell-Type Specific

To understand the relative contributions of TLR or Dectin receptor activation, the individual cytokine concentration data were assessed and the ratio of the remaining signal was modeled by fitting a linear model on the log-transformed data. In all cases, data obtained using NF-κBi were considered maximal inhibition. Overall, classical CD14^+^ monocytes had a mildly reduced cytokine response following TLR or Dectin inhibition (Figure 6). Inhibition of both Dectin and TLR led to a response reduction of the order of the sum of the reductions induced by single inhibitors (on the log-transformed data). Neutrophil responses, on the contrary, showed slight TLR dependence and high Dectin dependence (Figure 6); and the cytokine response of CD2^+^ cells was TLR dependent (25–80% inhibition) and poorly Dectin dependent (<25% inhibition, Figure 6). Notably, we found no evidence for a synergistic effect (*q* > 0.05 for all analytes and populations, see Materials and Methods). Additionally, we observed that for all cell types and most cytokines, inhibition of NF-κB had a significantly greater impact than combined inhibition of TLR2&4 and Dectin-1&2. This analysis suggests that there are additional sensors engaged by BCG, which contribute to the signaling through NF-κB.

### Induced Cytokines Amplify the Response to BCG

To parse the immune activation achieved by direct engagement of host sensors *versus* the feed-forward action of induced cytokines, we next conducted blocking studies aimed at limiting the downstream effects of key cytokine pathways: IL-1α/β, IL-6, and TNF-α. We first evaluated the expression of these cell surface receptors in whole-blood cell populations (Figure S7 in Supplementary Material). Whole-blood samples from healthy donors were stimulated by BCG in the combined presence of IL-1RA (the clinically approved molecule Anakinra), anti-CD130 (the gp130 subunit of IL-6R), and anti-CD120a (the p55 subunit of the TNFR). Of note, dose titration study confirmed the pharmacodynamics effect of IL-1RA (Figure S8 in Supplementary Material). Simultaneous blockade of these three receptors resulted in a significant reduction of the BCG protein signature (ranging from 22% for IL-8 to 60% inhibition for IL-1α and IL-1β; Figures 7A,B). The one exception from the list of measured analytes was MIP-1α, which showed a highly variable response to cytokine receptor blockade. Notably, inhibition of single receptors showed minimal impact on BCG-induced inflammatory responses, thus suggesting redundancy in the feed-forward action of induced cytokines (Figure 7B). We next utilized the same strategy on purified monocytes. The data obtained confirmed the observations using whole blood, showing a significant blunting of the BCG protein signature when cells were exposed to a combination of the three receptor blocking reagents as compared to control conditions (Figure 7C). These results suggested that cytokine receptor-mediated amplification of the response to BCG accounted for 20–60% of the observed inflammatory response, even in cell types expressing host sensors that are directly engaged by BCG agonists.

Several cytokine receptors signal *via* engagement of Janus kinases (JAKs), key regulators of signal transduction pathways (27). Importantly, JAKs are not known to mediate signaling downstream of host sensors (e.g., TLR and CLR pathways). Therefore, we tested the role of feed-forward cytokine receptor signaling in BCG responses by blocking JAK1/2 using INCB0184224 (also known as Ruxolitinib, clinically approved for the treatment of myelofibrosis, and referred to here as JAKi). Of note, we confirmed surface expression of the JAK1/2-dependent IL-6 and IFN-γ receptors on immune cell populations (Figure S7 and Table S2 in Supplementary Material). We measured the impact on cytokine and chemokine expressions, showing that at the highest dose of JAKi, we observed a considerable inhibition of the inflammatory response induced by BCG stimulation. In whole blood and purified CD14^+^ monocytes, 20–30% inhibition of MIP-1α and MIP-1β was observed; and 40–50% inhibition of IL-6, TNF-α, and IL-1β (Figures 7D,E; Figure S9 in Supplementary Material). Similar results were observed in neutrophils with a significant reduction of MIP-1α, MIP-1β, and IL-8 (15, 50, and 35%, respectively) and in CD2^+^ lymphocytes, where JAK inhibition resulted in a dose-dependent inhibition of IL-6, TNF-α, MIP-1α, MIP-1β, IL-1β, and IFN-γ (Figures 7F,G; Figure S9 in Supplementary Material). Strikingly, TNF-α and IFN-γ productions by lymphocytes were dramatically impacted by JAKi, showing 80–100% diminished signals, while IL-8 production was unchanged despite increasing concentrations of JAKi. The significant impact of JAKi is consistent with its profound suppression of inflammation observed in diverse pathological situations (28, 29). These results provide conclusive evidence for the critical involvement of cytokine receptor signaling in the modulation of BCG-induced inflammation.

**Figure 8:**
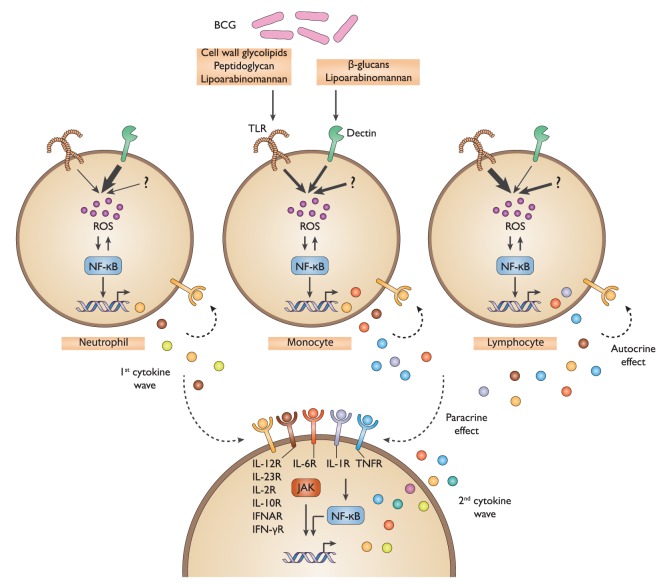
Schematic representation of the defined bacillus Calmette–Guérin (BCG) protein signature. Multiple BCG component agonists activate TLR2&4 and Dectin-1&2, host sensors that are expressed on diverse immune cells. This triggers the activation of ROS production and NF-κB signaling, resulting in the induction of a first wave of cytokines and chemokines. In turn, these stimulatory host factors act to amplify the response in an autocrine and paracrine manner through a combined activation of cytokine receptors, specifically IL-6R, IL-1R, and TNFR, as well as others, and the subsequent NF-κB and JAK-mediated signaling, thereby promoting the release of a second wave of cytokines and chemokines. TLR, toll-like receptor; ROS, reactive oxygen species; NF-κB, nuclear factor-kappa B; JAK, Janus kinase.

In summary, our work has detailed the cellular and molecular mechanisms of action by which BCG stimulates cytokine and chemokine production by immune cells. We demonstrated that the BCG protein signature results from the combined activation of multiple PRRs across diverse immune cell populations with an amplification of the signal achieved by autocrine and paracrine cytokine receptor signaling (Figure 8).

## Discussion

The discovery of PRRs triggered two decades of intense investigation into microbial stimuli and host sensors, which govern induced inflammatory responses (30). We have approached this problem from a systems immunology perspective, aimed at deconvoluting the mechanism of induced inflammatory responses triggered by a complex microorganism that is capable of engaging multiple immunological receptors, expressed by diverse cell types. Our study provides new insights into the coordinated BCG-induced inflammatory response, clarifying the specific role of TLR and Dectin activations, as well as the contribution of induced cytokines that act to amplify the inflammatory signature in whole blood. Briefly, we established CD14^+^ monocytes as the major source of innate cytokines and chemokines, with a unique and surprisingly rapid protein expression profile that is mediated by TLR2&TLR4 and Dectin-1&2 engagements. With respect to the direct engagement of other cell populations, we find a distinct pattern of sensor engagement, with our data suggesting that by contrast to monocytes, neutrophils respond to BCG primarily *via* Dectin-1&2, whereas lymphocytes utilize primarily TLR2&TLR4. While additional mechanistic studies are required, we suggest that engagement of host sensors may depend on the phagocytic activity or variable phagosomal pH, thus explaining the observed differences between cell types that share similar receptor expression (31, 32).

Applying multiple linear regressions, we modeled the experimental results and determined that engagement of TLR2&4 and Dectin-1&2 contributes to the induced protein signature in an additive fashion. Interestingly, this observation does not support the synergistic interactions described when using combinations of purified synthetic ligands (33–35). One possible explanation is that, in the context of BCG stimulation, TLR and Dectin receptor signaling converges on IκK kinase, acting *via* an MyD88/TIRAP scaffold or the Malt1/BCL10/Card9 complex, respectively (36).

When comparing the overall inhibition achieved by TLR2&4 and Dectin-1&2 with that observed when inhibiting NF-κB, we conclude that additional ligand/host sensor interaction(s)/or an undefined sensor participate in the overall response to BCG. In considering the extensive set of mycobacteria-associated molecular pattern molecules, this is not unexpected. It is well known that the bacterial cell wall contains multiple ligands for TLR and CLRs. Potential host sensors, such as DC-SIGN and mannose receptor, may contribute to the recognition of BCG and the induced NF-κB-mediated inflammatory response, although this remains to be tested. Additionally, there is evidence that endosomal and cytosolic sensors are engaged by mycobacterial nucleic acids (37, 38).

In addition to characterizing the role of host sensors, we uncovered a critical role for cytokine receptors and their subsequent signaling pathways, which substantially contribute to the complex response to BCG. Remarkably, within 3 h of exposure to BCG, we observed the secretion of robust levels of TNF-α, IL-6, and IL-1β. As such, these and likely other cytokines contribute *via* positive feedback amplification loops that augment NF-κB activation as well as engage JAK/STAT cytokine receptor signaling pathways. Interestingly, the response to cytokine stimulation alone did not predict its profound impact on the global BCG protein signature. Soumelis and colleagues provided some insight into this observation: taking a combinatorial approach, they evaluated the impact of co-culturing microbial stimuli in the presence of various cytokines. They report multiple patterns of cross talk with differential effects on human monocytes and pDCs (39). Additionally, our recent work has highlighted that in conditions of limiting dose of microbial stimulus, cytokines participate in the spreading of the inflammatory signal. Moreover, they report that the core cytokine-induced signatures are capable of capturing the variable response to stimulation by complex microorganisms (40). Together, our strategy enabled detailed analysis of the response to BCG with the demonstration that a diverse set of immune cells underlay what is a plastic and flexible inflammatory response. Such complexity may have evolved as a means to tailor the inflammatory response to the type, the infectious dose, and route of entry for a given microbe.

Our study also highlights the untapped opportunity to harness inflammatory signatures for immune monitoring. Growing consideration for systems biology approaches to monitor dynamic immunological changes demands standardized and well-characterized methods for immune stimulation. We propose that capturing the complexity of the host response to BCG will more accurately represent the variability of responses found in human health and disease states. Current efforts are aimed at investigating if complex stimuli can help to discriminate active from latently infected TB patients, a major unmet public health priority. It is our supposition that with improvements in sample collection procedures and standardized protein analyte testing, it will be possible to extend the diagnostic use of induced mycobacterial inflammatory responses. Regarding bladder cancer and BCG immunotherapy, our recent work provided direct evidence that parenteral BCG vaccination, prior to intravesical BCG instillation, accelerates host tumor immunity (41, 42). This and other observations in the field (43) have reinforced the potential diagnostic use of BCG-induced responses as an *ex vivo* diagnostic biomarker for stratifying patients prior to intravesical therapy.

In sum, we proposed a method of systems-level deconvolution of the BCG response by different factors, including time, cell type, and signaling pathway, which might be relevant for other complex stimuli and patients’ categories. Such characterization of the response will likely provide biomarkers, which may ultimately be applied to help to predict immunotherapeutic response in bladder cancer and support diagnosis of TB infection.

## Ethics Statement

Human samples: data presented in Figures 1 and 2 are obtained using samples from the Milieu Intéreur Healthy Donor Cohort. Details are provided in (11) and information about the cohort was registered at http://www.clinical-trials.gov (identifier NCT01699893). Data presented in Figures 3–7 were obtained using fresh whole blood from healthy volunteers, collected in heparin coagulant tubes and procured from Etablissement Français du Sang (EFS). The collection protocols were designed and conducted in accordance with the ethical principles of the Declaration of Helsinki, and Good Clinical Practices as outlined in the ICH Guideline for Good Clinical Practices.

## Author Contributions

AB performed all *in vitro* experiments. JB performed the kinetic study as well as statistical analyses, with the help of MF. DD, LQ-M, and MA participated in the generation of data from healthy donors recruited for the LABEX Milieu Intérieur. AB and MA wrote the manuscript with the input of all authors.

## Conflict of Interest Statement

MA is an employee of Genentech and using standardized whole-blood stimulation systems in the monitoring of patients enrolled in cancer immunotherapy clinical trials. All other authors declare that they have no conflict of interest.
