# Supplementary material for: Deconvolution of the Response to Bacillus Calmette–Guérin Reveals NF-κB-Induced Cytokines As Autocrine Mediators of Innate Immunity
Source: Front Immunol. 2017 Jul 13;8:796. doi: 10.3389/fimmu.2017.00796 (PMC5507989; doi:10.3389/fimmu.2017.00796)
Supplement: Supplementary file 1 [file Data_Sheet_1.PDF]

Supplementary Figure 1

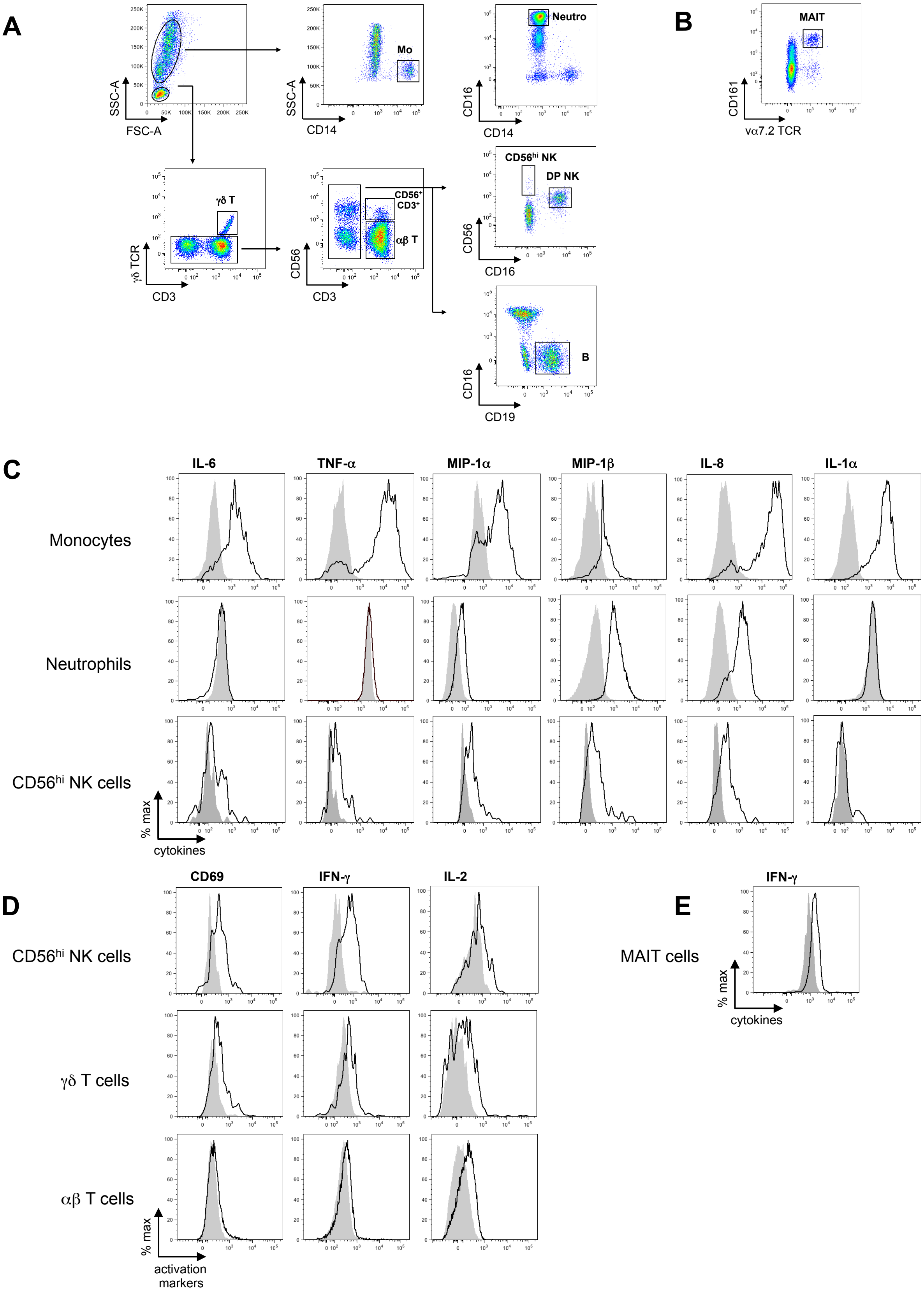

Supplementary Figure 1 – Strategy for mapping cell source of BCG induced proteins. Whole blood from >15 healthy donors was stimulated with BCG or buffer control for 16 h in the presence of Brefeldin A (BFA). Samples were stained with conjugated antibodies (**supplementary Table 2**) to discriminate the following cell populations: **(A)** $CD14^+$ monocytes (Mo), neutrophils (Neutro), γδ T cells (γδ T), ab T cells (αβ T), $CD3^+$ $CD56^+$ NKT-like cells ($CD3^+$ $CD56^+$), $CD56^{hi}$ NK cells ($CD56^{hi}$ NK), $CD16^+$ $CD56^+$ double positive (DP) NK cells (NKDP), and B cells, and **(B)** MAIT cells, in a second tube (panel #3). Following surface staining, the samples were permeablized and incubated with conjugated antibodies specific for intracellular cytokines and chemokines (**supplementary Table 2**). A dedicated gate for each intracellular protein was created for all the cell populations and cut-offs were established based on the negative control stimulation. **(C)** Representative intracellular staining for panel #1 is shown. **(D)** Representative intracellular staining for panel #2 is shown. **(E)** Representative intracellular staining of IFN-γ in MAIT cells in panel #3 is shown. BCG stimulated samples are indicated by a thick black line; unstimulated sample are shown in grey.

Supplementary Figure 2

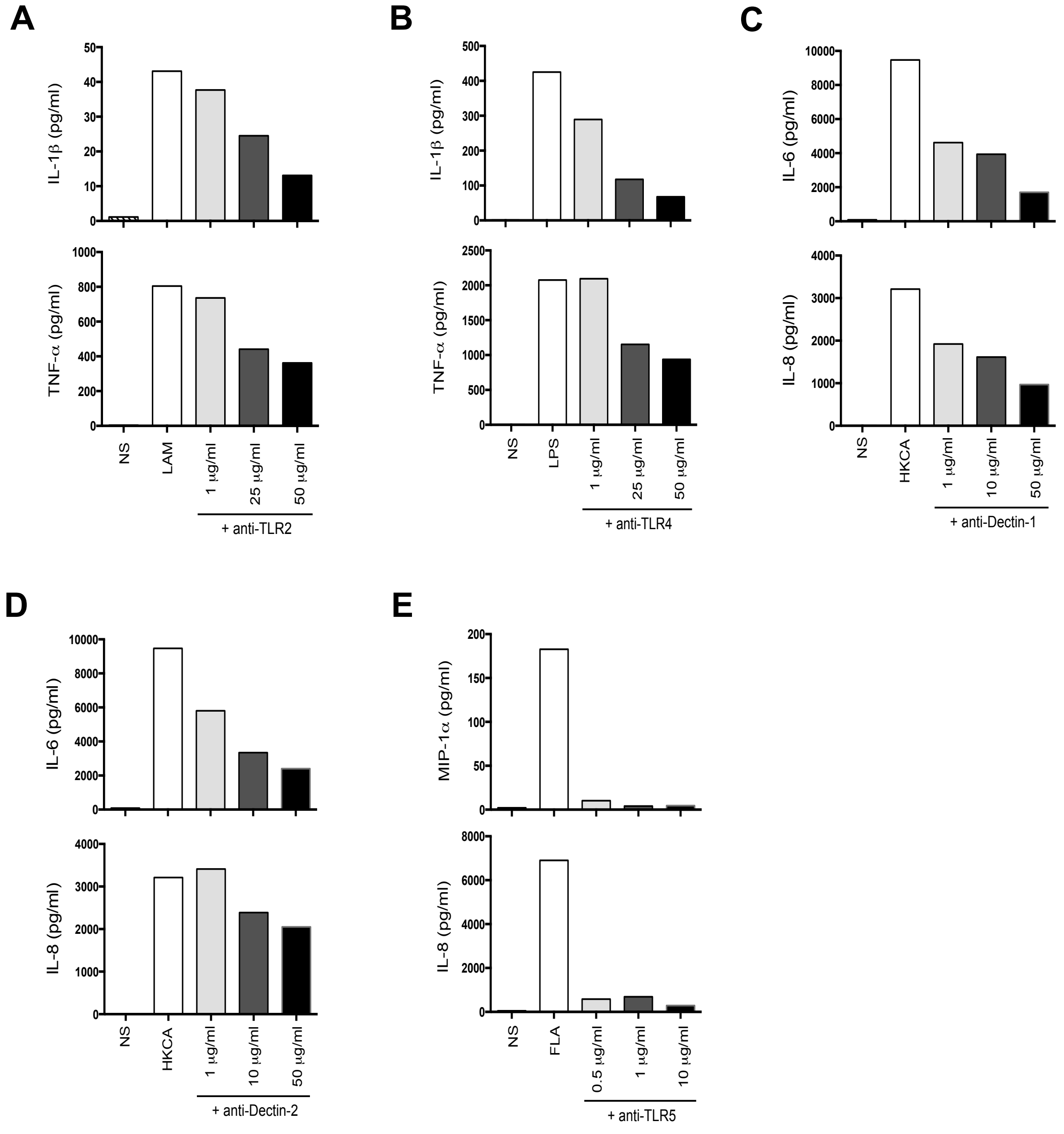

Supplementary Figure 2 – Dose titration study of neutralizing antibodies.

Whole blood samples were incubated for 5 h in the presence of increasing doses of neutralizing anti-TLR2 (A), anti-TLR4 (B), anti-Dectin-1 (C), anti-Dectin-2 (D), or anti-TLR5 (E). 1 h after the start of the incubation, the ligand of each receptor was added as followed: LAM, 10 mg/ml; LPS, 10 ng/ml; HKCA (Heat Killed preparation of *Candida Albicans*): 3x10^7 cells/ml; FLA (Flagellin from *Salmonella typhimurium*): 0.25 mg/ml. Cytokines concentration were measured with Luminex assay, and two representatives cytokines for each neutralizing condition were represented.

Supplementary Figure 3

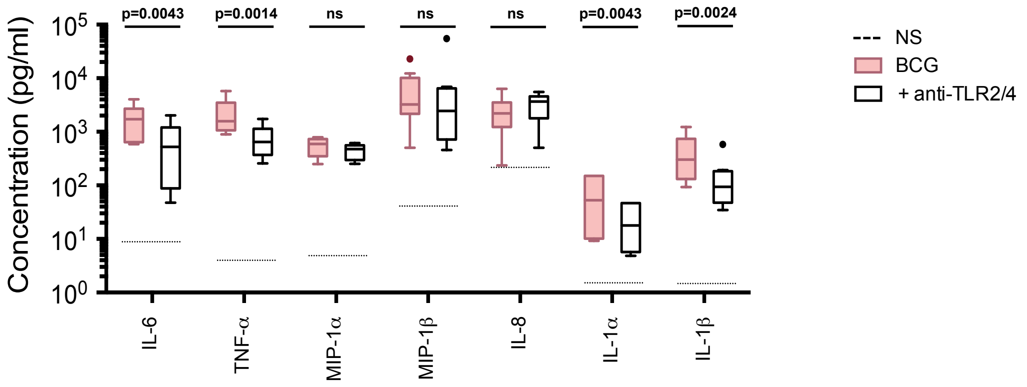

Supplementary Figure 3 – Impact of TLR2/4 neutralization on BCG-stimulated whole blood samples.

Concentrations of cytokines were determined by Luminex assay for whole blood (n=8) stimulated with BCG in the presence of istopye control antibodies, or neutralizing anti-TLR2&4. The dotted lines indicate the median value for unstimulated samples. ns : not significant. p values were determined by the paired Student's t test and FDR corrected for multiple analyte testing.

Supplementary Figure 4

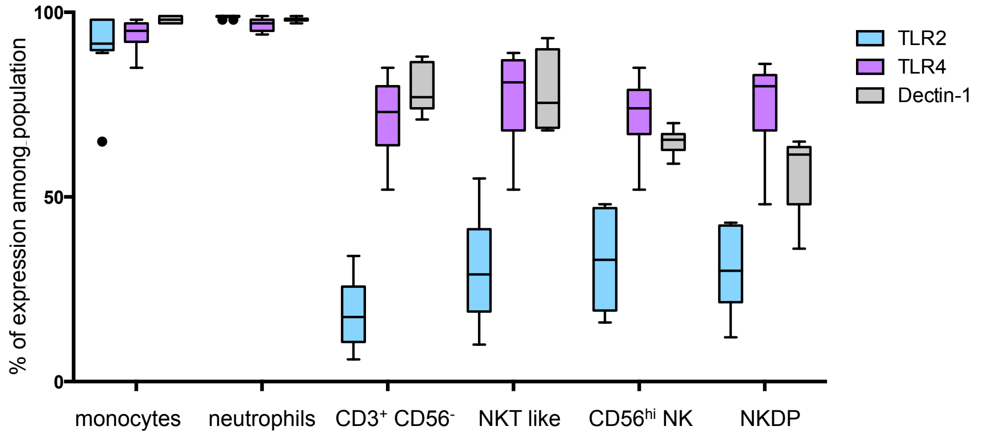

Supplementary Figure 4 – PRR expression on whole blood cell subsets.

Fresh whole blood from 6 to 9 donors was obtained and evaluated by flow cytometry for surface expression of TLR2, TLR4 and Dectin-1. The percentage of positive cells was determined using isotype control as a reference, and box-whisker plots indicate the expression across the donors tested.

Supplementary Figure 5

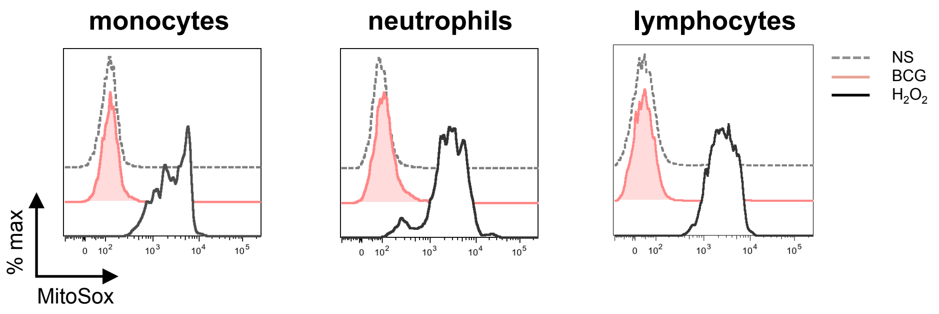

Supplementary Figure 5 – Detection of mitochondrial ROS using MITOSOX assay. Representative overlay of histograms of mitochondrial ROS measured on purified monocytes (n=4), neutrophils (n=4) and lymphocytes (n=4) stimulated for 4 h with BCG, media alone, or H_2O_2 using the MitoSox^{TM} Red reagent.

Supplementary Figure 6

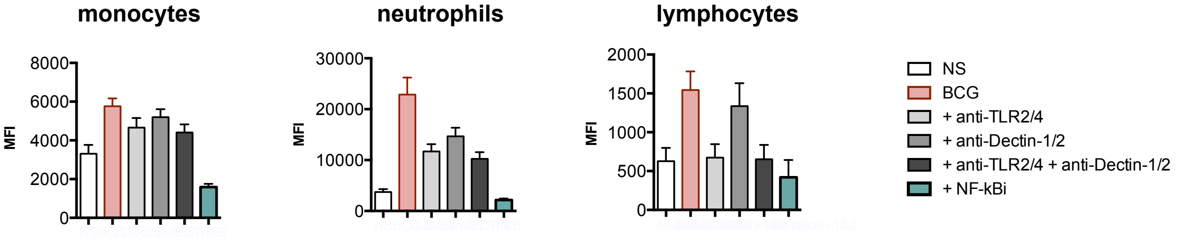

Supplementary Figure 6 – Histograms representation of total ROS detected with the DCFDA assay.

Intracellular ROS measured on purified monocytes (n=6), neutrophils (n=8) and lymphocytes (n=6) stimulated with BCG for a total of 5 h in the presence of neutralizing anti-TLR2&4, anti-Dectin-1&2, or anti-TLR2&4 + anti-Dectin-1&2, BCG plus isotype control antibodies, NF-κB inhibitor, or media control.

Supplementary Figure 7

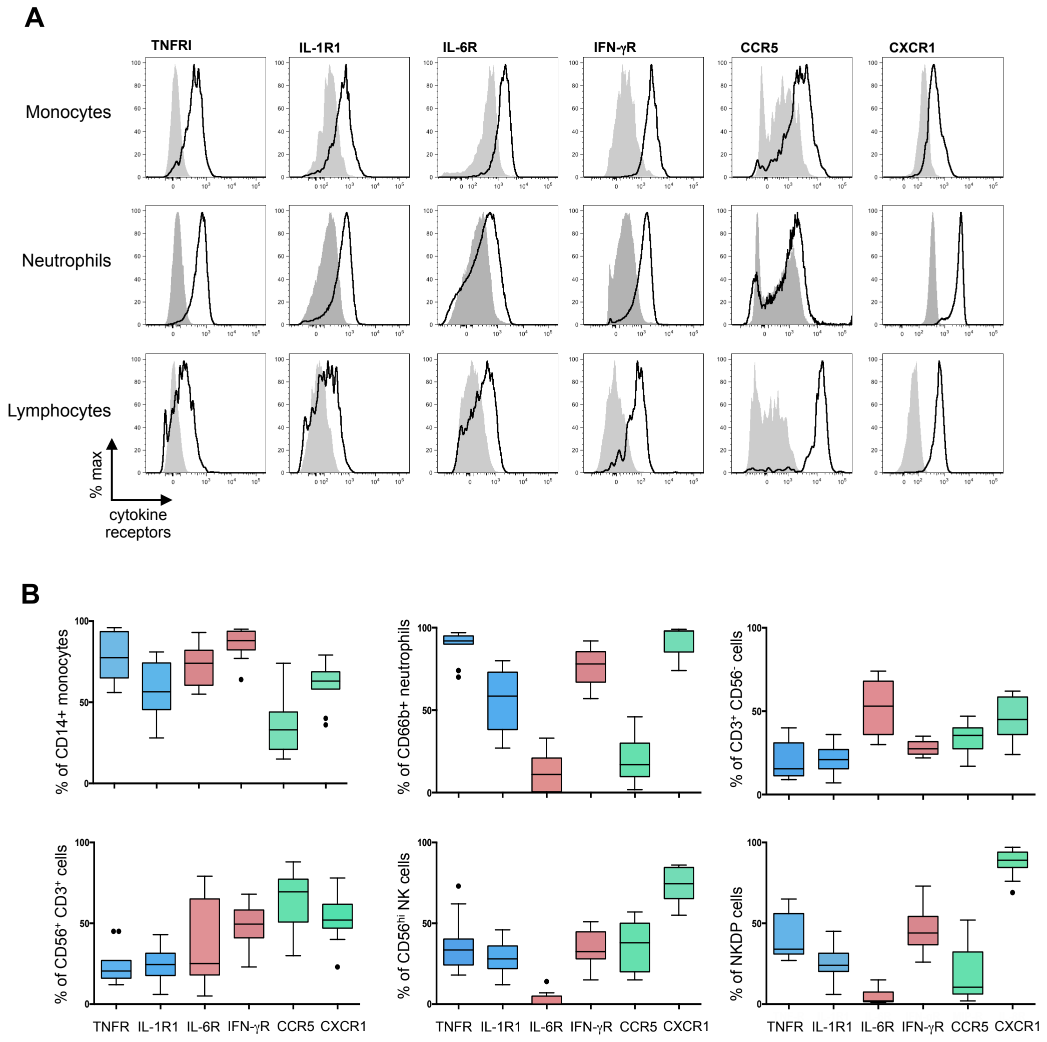

Supplementary Figure 7 – Surface expression of cytokines receptors on whole blood cell subsets

Fresh whole blood from 9 to 12 donors was obtained and evaluated by flow cytometry for surface expression of cytokines and chemokines receptors using specific conjugated antibodies (see supplementary Table 2 for more details). Corresponding isotypes controls were used to establish the gate cut-offs for each donor. **(A)** Representative surface staining for monocytes, neutrophils and lymphocytes population. Isotypes controls samples are shown in grey; samples stained with receptors-specific antibodies are indicated by a thick black line. **(B)** Tukey Box-whisker plots indicate the percentage of cytokine receptors positive cells for the six cell populations. Blue bars indicate NF-kB signaling dependent receptors, red bars JAK1/2 signaling dependent receptors, and green bars show chemokine receptors.

Supplementary Figure 8

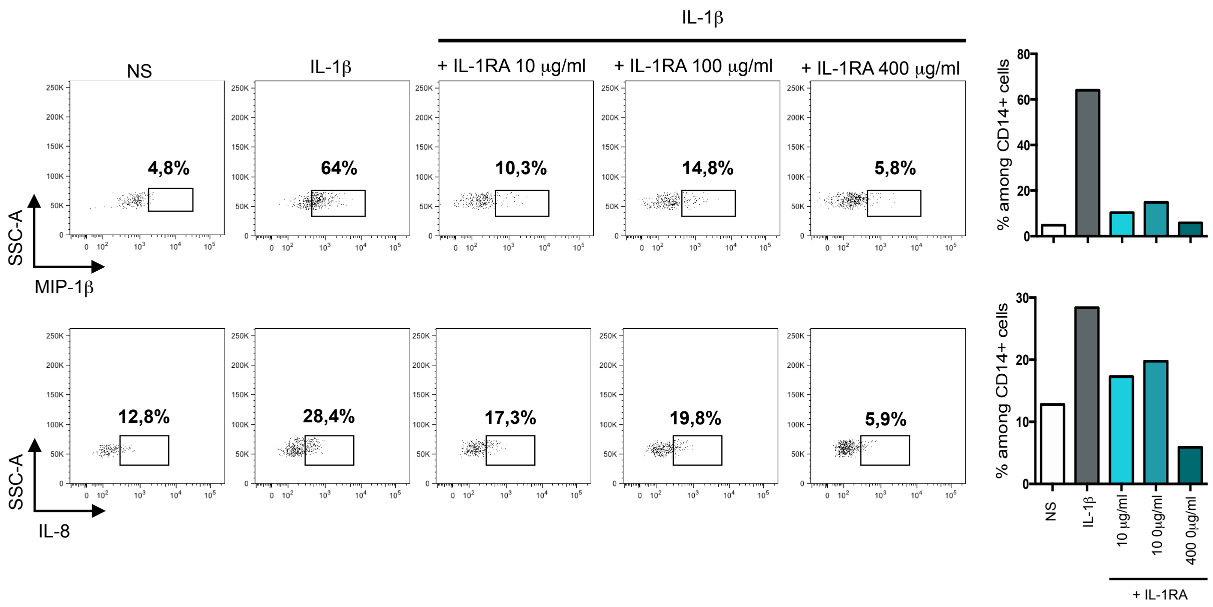

Supplementary Figure 8 – Dose titration study for IL-1RA (Kineret®).

Intracellular staining of MIP-1β and IL-8 from whole blood stimulated for 6 h with IL-1β in the presence of 10, 100 or 400 µg/ml of IL-1RA (added 1 h before). Brefeldin A was added at the same time as IL-1β. Debris were first removed and cytokines positive cells were determined on CD14+ monocytes. Dots plots are shown (left panel) and the data were represented in histograms (right panel) for both cytokines.

Supplementary Figure 9

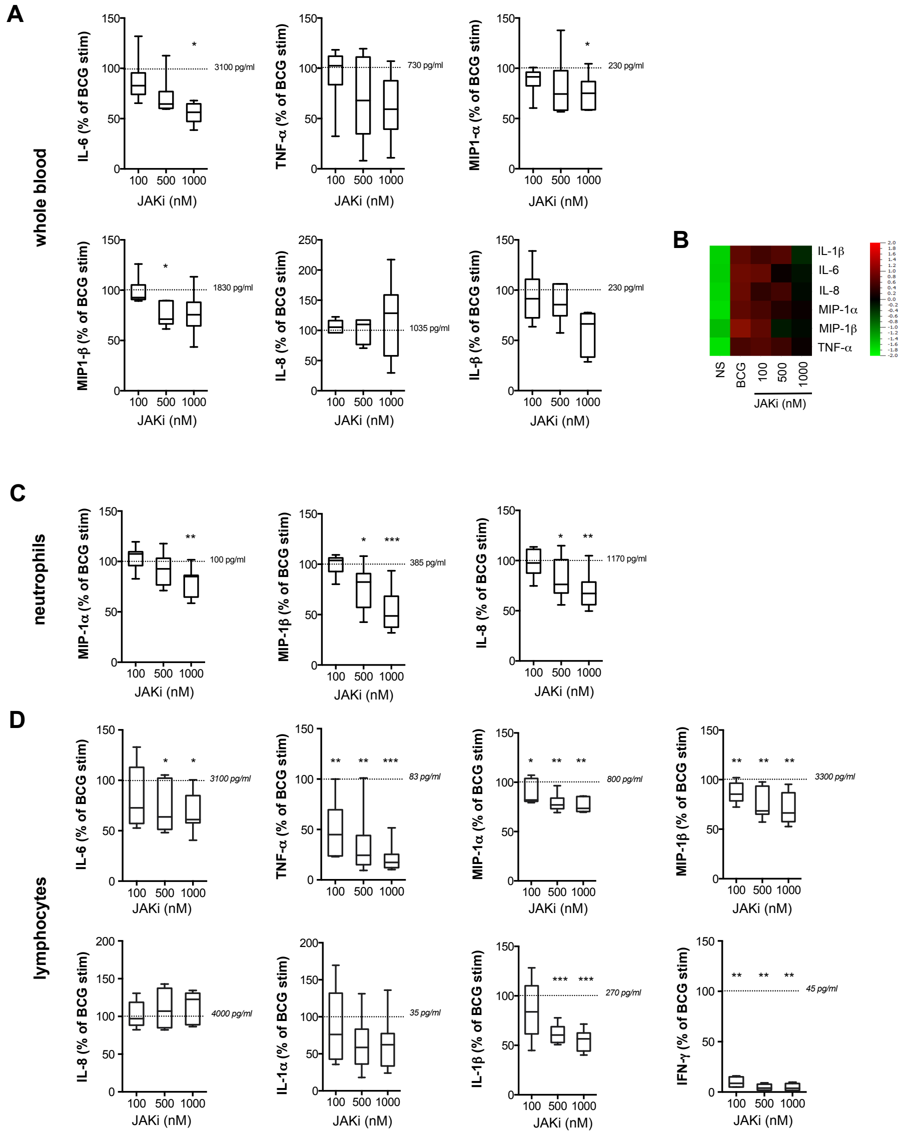

Supplementary Figure 9 – JAK1/2 involvement in BCG inflammatory response for whole blood, purified neutrophils and lymphocytes.

Whole blood (**A, B**, n = 6) or purified neutrophils (**C**, n= 9) or lymphocytes (**D,** n = 7) were stimulated by BCG in the presence of increasing doses of the JAK1/2 inhibitor Ruxolitinib. Box-whisker plots indicate the percentage inhibition as compared to BCG stimulation (normalized to 100% across experiments). The dotted lines indicate the median values for BCG stimulation for each cytokine. **(B)** Heat map represent the median concentration determined from the data showed for whole blood samples. p values were determined on the proteins concentration data sets using the paired Student's t test and FDR corrected for multiple analyte testing. *q≤ 0.05; **q≤ 0.01; ***q≤ 0.001.

Supplementary Table 1

Analytes	Abbreviation	Units	LDD*	LLOQ†
Brain-Derived Neurotrophic Factor	BDNF	pg/mL	23	36
Eotaxin-1	Eotaxin-1	pg/mL	10	145
Factor VII	Factor VII	pg/mL	6200	3800
Granulocyte-Macrophage Colony-Stimulating Factor	GM-CSF	pg/mL	16	88
Intercellular Adhesion Molecule 1 (soluble)	sICAM-1	pg/mL	1800	1300
Interferon gamma	IFNG	pg/mL	2	2
Interleukin-1 alpha	IL-1A	pg/mL	1.5	2
Interleukin-1 beta	IL-1B	pg/mL	2	3
Interleukin-1 receptor antagonist	IL-1RA	pg/mL	49	95
Interleukin-2	IL-2	pg/mL	5	8
Interleukin-3	IL-3	pg/mL	5.7	16
Interleukin-4	IL-4	pg/mL	22	29
Interleukin-5	IL-5	pg/mL	5	13
Interleukin-6	IL-6	pg/mL	4	11
Interleukin-7	IL-7	pg/mL	11	9
Interleukin-8	IL-8	pg/mL	3	4
Interleukin-10	IL-10	pg/mL	5	7
Interleukin-12 Subunit p40	IL-12p40	pg/mL	80	280
Interleukin-12 Subunit p70	IL-12p70	pg/mL	30	49
Interleukin-15	IL-15	pg/mL	580	390
Interleukin-17	IL-17	pg/mL	6	5
Interleukin-18	IL-18	pg/mL	18	41
Interleukin-23	IL-23	pg/mL	610	400
Macrophage Inflammatory Protein-1 alpha	MIP-1A	pg/mL	41	42
Macrophage Inflammatory Protein-1 beta	MIP-1B	pg/mL	20	31
Matrix Metalloproteinase-3	MMP3	pg/mL	33	58
Matrix Metalloproteinase-9	MMP9	pg/mL	25000	40000
Monocyte Chemotactic Protein 1	MCP-1	pg/mL	23	45
Stem Cell Factor	SCF	pg/mL	92	116
Tumor Necrosis Factor alpha	TNF-A	pg/mL	14	23
Tumor Necrosis Factor beta	TNF-B	pg/mL	11	10
Vascular Endothelial Growth Factor	VEGF	pg/mL	62	34

* The least detectable dose (LDD) was determined as the mean + 3 standard deviations of 200 blank readings. Results below the LDD are more variable than results above the LDD.

† The LLOQ (Lower Limit of Quantitation) is the lowest concentration of an analyte in a sample that can be reliably detected and at which the total error meets CLIA requirements for laboratory accuracy. As the LLOQ and the LDD values are independent from each other, on occasion the LLOQ is lower than the LDD.

Supplementary Table 2

	surface staining		intracellular staining	
	antibodies name	clones	antibodies name	clones
panel #1 "innate"	CD3 APC-H7[1]	SK7	IL-6 v450[1]	MQ2-13A5
	CD16 PerCP-Cy5.5[1]	3G8	TNF-α AF700[1]	MAb11
	CD56 PE-Cy7[1]	B159	MIP-1α PE[2]	CR3M
	CD14 BV500[1]	M5E2	MIP-1β APC[1]	D21-1351
	CD19 BV711[1]	SJ25C1	IL-8 PE-CF594[1]	G265-8
	TCR γδ BUV395[1]	B1	IL-1α FITC[2]	364/3B3-14
panel #2 "lymphocyte activation"	CD3 APC-H7			
	CD16 PerCP-Cy5.5		CD69 FITC[1]	L78
	CD56 PE-Cy7		IFN-γ PE[1]	4S.B3
	CD19 BV711		IL-2 APC[1]	MQ1-17H12
	TCR γδ BUV395			
panel #3 "MAIT"			IL-6 v450	
	CD3 APC-H7		TNF-α AF700	
	CD161 BV510 [1]	DX12	IFN-γ PE	
	TCR vα7.2 FITC[3]	3C10	MIP-1β APC	
			IL-8 PE-CF594	
PRR expression	Fc block [5]			
	CD3 APC-H7[1]	SK7		
	CD16 PerCP-Cy5.5[1]	3G8		
	CD56 PE-Cy7[1]	B159		
	CD14 BV500[1]	M5E2		
	CD66b BV421[1]	G10F5		
	CD369/Dectin-1[1]	3G8		
	TLR2 FITC[3]	TL2-1		
	TLR4 PE[3]	HTA125		
CKR expression	Fc block [5]			
	CD3 APC-H7[1]	SK7		
	CD16 PerCP-Cy5.5[1]	3G8		
	CD56 PE-Cy7[1]	B159		
	CD14 FITC[1]	M5E2		
	CD66b BV421[1]	G10F5		
	CD195/CCR5 BUV737[1]	2D7		
	CD130/IL-6R PE-CF594[1]	AM64		
	CD120/TNFR APC[3]	3G7A02		
	CD119/IFN-γR BUV786[1]	GIR-208		
	CD181/CXCR1 BUV395[1]	5A12		
	IL-1R1 PE[4]	FAB269P		

Antibodies purshased from BD Biosciences [1], Ebioscience [2], Biolegend [3] ,R&D Systems [4], or Miltenyi [5]
